# Calibration and validation of toxicokinetic-toxicodynamic models for three neonicotinoids and some aquatic macroinvertebrates

**DOI:** 10.1007/s10646-018-1940-6

**Published:** 2018-05-01

**Authors:** Andreas Focks, Dick Belgers, Marie-Claire Boerwinkel, Laura Buijse, Ivo Roessink, Paul J. Van den Brink

**Affiliations:** 1 Wageningen Environmental Research, Wageningen University and Research, P.O. Box 47, 6700 AA Wageningen, The Netherlands; 2 Department of Aquatic Ecology and Water Quality Management, Wageningen University and Research, P.O. Box 47, 6700 AA Wageningen, The Netherlands

**Keywords:** Aquatic ecotoxicology, Time-variable exposure, Toxicokinetic-toxicodynamic modelling, Neonicotinoids, Macroinvertebrates

## Abstract

Exposure patterns in ecotoxicological experiments often do not match the exposure profiles for which a risk assessment needs to be performed. This limitation can be overcome by using toxicokinetic-toxicodynamic (TKTD) models for the prediction of effects under time-variable exposure. For the use of TKTD models in the environmental risk assessment of chemicals, it is required to calibrate and validate the model for specific compound–species combinations. In this study, the survival of macroinvertebrates after exposure to the neonicotinoid insecticide was modelled using TKTD models from the General Unified Threshold models of Survival (GUTS) framework. The models were calibrated on existing survival data from acute or chronic tests under static exposure regime. Validation experiments were performed for two sets of species-compound combinations: one set focussed on multiple species sensitivity to a single compound: imidacloprid, and the other set on the effects of multiple compounds for a single species, i.e., the three neonicotinoid compounds imidacloprid, thiacloprid and thiamethoxam, on the survival of the mayfly *Cloeon dipterum*. The calibrated models were used to predict survival over time, including uncertainty ranges, for the different time-variable exposure profiles used in the validation experiments. From the comparison between observed and predicted survival, it appeared that the accuracy of the model predictions was acceptable for four of five tested species in the multiple species data set. For compounds such as neonicotinoids, which are known to have the potential to show increased toxicity under prolonged exposure, the calibration and validation of TKTD models for survival needs to be performed ideally by considering calibration data from both acute and chronic tests.

## Introduction

Environmental risk assessment procedures relate predicted exposure concentrations in environmental compartments to effect thresholds usually derived from standard toxicity tests. Exposure concentrations, e.g., in water bodies adjacent to agricultural fields, show highly variable dynamics, which depend on chemical application schemes and entry pathways (e.g., spray drift, surface runoff and erosion, or drainage (Bach et al. 2017; Wittmer et al. 2010). These field-relevant dynamic concentration patterns are generally not considered in the ecotoxicological effect assessment of chemicals. Instead, in standard toxicity tests a constant exposure to the chemical is maintained over a defined test duration. In consequence, metrics derived from toxicity tests do not reflect the risk that might result from realistic exposure patterns of the chemical. The discrepancy between having constant exposure in ecotoxicological experiments and dynamic exposure concentrations under field conditions can, next to laborious experiments with refined exposure profiles in the laboratory, be overcome by using toxicokinetic-toxicodynamic (TKTD) models. Such models account for the dynamics of external exposure and connect them to internal exposure accounting for uptake, depuration and damage processes over time. TKTD models are specifically supposed to deliver relevant information for the extrapolation from toxicity observed under static exposure conditions to expected effects under time-variable exposure in the context of environmental risk assessment of pesticides (Ashauer and Escher 2010). TKTD models from the family of General Unified Threshold models of Survival (GUTS) have the potential to calculate survival probabilities for any time-variable exposure profile (Jager et al. 2011). GUTS models are structurally simple and consist of three or four equations, that capture species- and compound-specific information in the model parameters. The model parameters have to be determined from observed survival data for each species and compound combination separately. Recently, it was reported that standard survival test results could be used for the parameterisation of TKTD models for the fungicide propiconazole and *Gammarus pulex*, without losing accuracy of model predictions (Nyman et al. 2012). If raw data from standard (acute or chronic) toxicity tests is sufficient in the predictive capacity also for other species-compound combinations, the application of GUTS models will be eased considerably, because laborious and expensive measurements of internal concentrations won’t be required for model calibration.

For an application in regulatory ERA, it is not sufficient to calibrate model parameters and generate predictions of survival, but it is required to test the quality of model predictions, or with other words, to validate the model for specific combinations of species and compounds. Validation in a broader context, for example for the evaluating of a complex population- or community model application, requires a comprehensive approach, which Augusiak and colleagues termed ‘Evaludation’ (Augusiak et al. 2014). In the context of the relatively simple survival modelling as done with GUTS, validation means to compare model output to new data that were not used for model parameterisation, a process called ‘output corroboration’. Validation studies in a risk assessment context are required amongst others by the European Food Safety Authority (EFSA) as prerequisite for the acceptability of ecological modelling studies for regulatory risk assessment (EFSA 2014). Validation studies enable also to evaluate the validity of the modelling assumptions and, hence, to test the predictive power of model simulations. Considering the extrapolation of mortality from constant to time-variable exposure patterns, survival data from experiments using time-variable exposure appear most meaningful and relevant for validation purposes. Some examples of studies that tested TKTD model predictions consider e.g., the combined effects of carbaryl and chlorpyrifos on *Gammarus pulex* (Ashauer et al. 2007a), the effects of pulsed exposure to pentachlorphenol and chlorpyrifos on *G. pulex* (Ashauer et al. 2007b*)*, and the effects of propiconazole on *G. pulex* (Nyman et al. 2012).

In this study, we present validation results for GUTS models for five aquatic macroinvertebrate species and three neonicotinoid insecticides, i.e., imidacloprid, thiamethoxam and thiacloprid. Neonicotinoid compounds are recently under intense discussion since they have been reported to occur frequently and in concentrations up to effective levels in surface waters in Europe (Morrissey et al. 2015; Vijver and Van den Brink 2014). In addition, neonicotinoids receive special attention because they were reported to affect mobility and survival of aquatic macroinvertebrates already at low concentrations (Roessink et al. 2013) and are additionally suspected to show reinforced toxicity with longer time of exposure (Tennekes and Sánchez-Bayo 2013). Two different sets of experiments have been performed to investigate the effect of time-variable concentrations of neonicotinoids on the survival of aquatic macroinvertebrates and hence to deliver validation data for model predictions. Table 1 gives on overview about tested compounds, species, and the type of exposure. The first set of experiments focussed on multiple species and tested the effect of pulsed exposure profiles of imidacloprid on the survival of *Asellus aquaticus*, *Caenis horaria*, *Cloeon dipterum*, *Chaoborus obscuripes* and *Plea minutissima*. A second set of validation experiments focussed on multiple compounds, and investigated the effects of pulsed exposure profiles of imidacloprid, thiacloprid and thiamethoxam on the survival of *C. dipterum*.

**Table 1:** Overview of experiments used for model calibration and validation

Data set	Compounds	Tested species	Start of experiments	Exposure	Reference
Multiple species (MS)					
Calibration					
MS_C1	Imidacloprid	*Asellus aquaticus*, *Caenis horaria*, *Chaoborus obscuripes*, *Cloeon dipterum*, *Plea minutissima*	22/09/2014 17/11/2014 06/10/2014 06/10/2014 06/10/2014	Acute–static (4 days)	(Van den Brink et al. 2016)
Validation					
MS_V	Imidacloprid	*Asellus aquaticus*, *Caenis horaria*, *Chaoborus obscuripes*, *Cloeon dipterum*, *Plea minutissima*	16/09/2014 27/11/2014 11/11/2014 04/11/2014 19/11/2014	3 pulsed regimes (between 21–28 days)	This study
Multiple compounds (MC)					
Calibration					
MC_C1	Imidacloprid Thiacloprid Thiamethoxam	*Cloeon dipterum*	April 2013	Acute–static (4 days)	(Van den Brink et al. 2016)
MC_C2	Imidacloprid Thiacloprid Thiamethoxam	*Cloeon dipterum*	24/02/2015	Chronic–semi-static (28 days)	This study
Validation					
MC_V	Imidacloprid Thiacloprid Thiamethoxam	*Cloeon dipterum*	17/03/2015	2 pulsed regimes (28 days)	This study

The first aim of this study was to analyse whether we can predict the survival of macroinvertebrate individuals under exposure to time-variable concentrations of different neonicotinoid compounds by using GUTS models calibrated only on standard toxicity test data with a sufficient accuracy. A second aim was to analyse the influence of model type (stochastic death (SD) vs. individual tolerance (IT)), calibration data set, and the choice of compound and species on the quality of predictions. For the multiple compound dataset, GUTS models were calibrated based on both acute or chronic toxicity test results and corresponding predictions of the survival over time were calculated to evaluate which model fits better to the observed survival.

## Material and methods

Survival data from a number of acute or chronic tests performed using static exposure regimes (calibration experiments; Table 1) were employed to calibrate GUTS versions applying the scaled internal concentration as dose metric (Jager et al. 2011). Calibrated models were used to predict survival under time-variable exposure using measured external concentrations from the validation experiments as input. Resulting predicted survival was compared with the observed survival in the validation experiments (Table 1).

### Data

#### Calibration data sets

Survival of tested invertebrates and measured concentration data from previously published acute experiments (Table 1) under static or semi-static exposure were used for model calibration (Van den Brink et al. 2016). These experiments were all performed using five concentrations and a control, each having at least three replicates. In experiment **MS_C1**, acute tests were performed with imidacloprid and winter generations of five aquatic macroinvertebrate species. Survival of the individuals after dosing the systems at day 0 was observed daily for 4 days. In experiment **MC_C1**, the winter generation of *C. dipterum* was tested for acute toxicity of imidacloprid, thiacloprid and thiamethoxam. After dosing of the systems at day 0, living individuals were kept in these systems until day 4, and counted daily. More details about these data sets are given in the original publication (Van den Brink et al. 2016).

Additionally to these published data sets, results from an additional chronic test (experiment **MC_C2)** evaluating the effects of three neonicotinoid insecticides on a winter generation of *C. horaria* were used for model calibration, where five treatment levels were tested in triplicates and nine control replicates were included (Table S1). Every week (T = 7, 14 and 21), the test systems were completely refreshed including renewal of the chemical (semi-static design), and survival of the individuals was monitored. Concentrations of the test compounds were measured in all treatments before and after refreshment of the systems (see Neonicotinoid application, sampling and analysis). Each replicate test system contained 10 *C. dipterum* individuals. Test animal collection was according to Van den Brink et al. (2016). During acclimatization and test periods, animals were fed on species specific food, i.e., a combination of conditioned organic matter (four small rounds (ø 14 mm) of populous leaves), periphytic algae and *Elodea nuttallii* (top shoot, 5 cm). Deep groundwater from a well at the Sinderhoeve test facility, collected 5 days before dosing, aerated for at least 24 h before animals were added, was used as test water. More details about experimental conditions are given in ESM2.

#### Validation data sets

Two separate sets of experiments on the survival of aquatic macroinvertebrates under time-variable exposure were performed. The experiments have been performed in parallel to the acute test with the same choice of species (Table 1). Test animals were collected at the same locations and kept under same conditions as described in Van den Brink et al. (2016).

In experiment **MS_V**, the survival of individuals of five aquatic macroinvertebrate species, *Asellus aquaticus, Caenis horaria, Chaoborus obscuripes, Cloeon dipterum* and *Plea minutissima*, was tested under different time-variable (pulsed) exposure profiles of imidacloprid. Per jar, 15 individuals were tested in 4 treatments with 5 replicates each. The treatments included a control, and three different pulsed exposure profiles with pulse concentrations representing the LC_30_ (48 h) as determined from acute tests. In treatment P1, the test systems were dosed once on day 0, individuals were transferred to clean water on day 2, and stayed there for the remainder of the test period. In treatment P2, systems were dosed on days 0 and 7, while in P3 dosing occurred on day 0 and day 14. In both tests, test organisms were transferred to clean water 24 h after dosing, hence the total exposure duration was the same for P1, P2 and P3. For *P.minutissima*, however, all three treatments were dosed a third time at day 21. Individuals of all treatments (including controls) which were not dosed with the pesticide were transferred into fresh clean water at days 2, 7, 8, 14, and 15 to subject all test organisms to the same handling conditions for all treatments. Samples for residue analysis have been taken in all treatments before and after refreshment of the systems to ensure correct exposure. Samples were stored frozen awaiting further analysis. Living individuals were counted on 15 observation days.

In experiment **MC_V**, the survival of *Cloeon dipterum* under two different pulsed exposure regimes to imidacloprid, thiacloprid and thiamethoxam was investigated. For the toxicity testing, per jar 10 individuals were tested in 3 replicates per compound and tested profile. In additional controls, the survival of *C. dipterum* without exposure was observed using 9 replicates. The treatments included two pulsed exposure regimes with pulse heights equalling the LC_30_ (48 h) and with a duration of 24 h each. In both treatments, dosing occurred on day 0, and after 24 h the individuals were transferred into systems containing clean water. In treatment P1, individuals were dosed a second time on day 7, while in treatment P2 a second dosing occurred on day 14. Samples have been taken from treatments before and after refreshment of the systems for residue analysis. Individuals of all treatments (inclusive controls) were transferred into fresh water on days 2, 7, 8, 14, 15. Living individuals were counted on in total 9 observation days (days 1, 3, 7, 10, 14, 17, 21, 24, 28). During the 24 h exposure periods, test organisms were housed in test systems without food (e.g., biofilm, *Populus* leaves and *Elodea*) to avoid any interference with the planned exposure pattern by for instance sorption of the test compound to organic matter. 1 day after each dosing (on day 1, 8 and 15) animals were transferred to clean test medium containing sufficient food. All other experimental conditions were identical to the chronic tests (experiments MC_C2).

#### Neonicotinoid application, sampling and analysis

For the experiments MC_C2 and MC_V, dosing stock solutions for IMI, TC, and TM were prepared using a water soluble granule formulation containing 70% IMI w/w, a suspension concentrate formulation containing 480 g TC/L, and water dispersible granules containing 25% TM w/w, respectively. For the MS_V experiments, technical grade IMI (Dr Ehrenstorfer Gmbh) was used for a stocking solution. From these stock solutions dosing solutions were prepared and added to copper free tap water in the jars to achieve the desired nominal concentrations. Samples were taken from the dosing solutions to confirm neonicotinoid exposure concentrations. The 1 μg/L treatment of thiamethoxam in the MC_C2 data set was wrongly dosed on day 21, when the 10-fold of the intended dose was applied.

For sampling, aliquots of approximately 3 mL were collected with a glass Pasteur’s pipette and transferred into 4 mL glass vials containing 1 mL of acetonitrile, shaken thoroughly by hand, and subsequently stored in a freezer at −20 °C prior to analysis. The samples with an expected concentration > 1 μg a.i./L were analysed by direct injection on LC-MS/MS (Agilent Technologies). The samples with an expected concentration ≤ 1 μg a.i./L were concentrated by percolating 100 mL sample over a solid phase extraction (SPE) column and washing the columns with 2*2 mL of acetonitrile and suspended in 1 mL of copper free tap water. The samples were subsequently analysed by LC-MS/MS.

The complete exposure data as measured in experiments MC_C2, MC_V and MS_V are given in the electronic supporting material ESM1.

### Modelling

The General Unified Threshold models of Survival (GUTS) framework was used (Jager et al. 2011). More specifically, parameters of the stochastic death (SD) and individual tolerance (IT) models in connection with the scaled internal concentration (SIC) were calibrated. These two model variants differ with respect to the variability of the assumed internal threshold within a group of individuals: in the SD model the threshold is assumed to be the same for all individuals within a group, and death occurs with a certain rate when this threshold is exceeded. In the IT model, death occurs immediately when an individual internal threshold is exceeded. The model equations have been documented earlier (Jager et al. 2011) but are given in the supporting information for comprehensiveness (ESM2). The GUTS model was implemented in Mathematica (Wolfram Research, version 11), and the implementation was tested recently in a ring test for correct implementation (Jager and Ashauer 2018).

#### Model calibration

Model calibration was done using survival counts and concentration measurements of the external exposure from the acute or chronic tests per replicate, if not stated differently, to account for differences in the exposure levels between replicates. The driving factor for modelling the toxicity was the external concentration *C*_ext_(t). The scaled internal concentration *C*_i_*(t) was used as a dose metric (eq. S1) and linked to the stochastic death (SIC-SD) or individual tolerance (SIC-IT) model to describe the survival rates over time (eq. S3 and S5). To find an optimal vector of parameter values Θ^opt^ for each of the SIC-SD and the SIC-IT models, parameter values were optimised with respect to match the experimentally observed survival data. Background mortality rates were fitted to the survival data in the controls separately. When no mortality occurred during the tests, the background mortality rate constant was set to 0.005 day^−1^_._ Survival rates for both the SIC-SD and the SIC-IT model were calculated in explicit dependence of the parameter vector $\theta$ and the measured external concentration over time (eq. S6 and S7). For the SIC-IT model, the dominant rate constant (k_D_), the median of the distribution of threshold values (α), and the related shape parameter (β) were calibrated, while for the SIC-SD model, the dominant rate constant (k_D_), the threshold (z) and the killing rate constant (k_k_) were calibrated. Survival in small cohorts follows a multinomial distribution, hence the log-likelihood function was applied for measuring the agreement between model and observations (eq. S8). Optimisation was done using the built-in optimisation routine *Simulated Annealing* of the method *NMinimize* of Mathematica (Wolfram Research, version 11.0). Parameter sets were obtained for the best fits between data and model simulations by minimisation of the negative log-likelihood function. More details about parameter optimisation can be found in the ESM (ESM2.2.2). The likelihood ratio method was used to estimate confidence intervals for the optimal parameter *Θ*^*opt*^ (Albert et al. 2012; Ashauer et al. 2016; Meeker and Escobar 1995). Confidence intervals for the single parameters and a given confidence level were numerically approximated. More details about the approach are given in the ESM (ESM2.2.3).

#### Prediction of survival under time-variable exposure profiles

In addition to structural uncertainty, i.e., the question whether a model structure is appropriate to describe observed dynamics, the main sources of uncertainty in model predictions are the stochasticity of the survival process in small cohorts of individuals, and the uncertainty of the estimated model parameters, which reflect the biological variability within the group of tested organisms. These uncertainties can be quantified and hence taken into account. We followed the approach as outlined in Ashauer et al. (2016). To account for biological variability as being captured in the parameter uncertainties, probabilistic predictions of survival were performed, where 1000 random parameter vectors from the parameter confidence regions were used. In addition, per parameter set, 10 repetitions of the stochastic survival process in a small cohort of individuals over time were performed, hence a total number of 10,000 simulations of survival over time were performed per exposure scenario and model type. More details are given in the ESM (ESM2.2.4). Probabilistic predictions over time were performed for the exposure patterns used in data sets MS_V and MC_V, resulting in predictions of the survival over time. In addition, the survival at the end of an experiment was calculated for a series of manipulated pulsed exposure profiles, resulting in a dose-response view on the model predictions. To create these exposure profiles, concentrations as measured in the original pulsed exposure experiments (MS_V and MC_V) were multiplied with a set of 61 multiplication factors, ranging from 10^−2.5^ to 10^3.5^. Predictions were performed following the same probabilistic approach as for the predictions of the survival over time (10,000 simulations per multiplication factor). Median and percentiles of the simulated survival at the end of the pulsed experiments were interpolated to create exposure profile specific simulated concentration response curves for survival at the end of the respective experiments.

#### Model validation

Model validation was done based on survival counts observed during the validation experiments (data sets MS_V and MC_V, see ESM5 (ESM5_raw-survival-data.ods). For validation, survival counts from the replicates per treatment were pooled to increase the size of the sample per treatment. Corresponding concentration measurements from the single replicates per treatment were averaged, which appeared valid because differences between the pulse concentrations were much larger than between the measured concentrations in the replicates.

A set of indicators for the accuracy of the model predictions were calculated for the survival over time. The model prediction error (MPE) as defined in Nyman et al. (2012), reports the mean relative deviation of predicted and observed survivors over time. A second criterion is based on the expectation that predicted and observed survival numbers match the 1:1 line in a scatter plot. The classical root-mean-square error (RMSE) aggregates the magnitude of the prediction errors for various time-points into a single measure of predictive power. In order to provide a criterion expressed as a percentage, the RMSE normalised by the mean of the observations was used:

1 $$NRMSE = \frac{{RMSE}}{{\bar Y}} = \frac{1}{{\bar Y}}\sqrt {\frac{1}{n}\mathop {\sum }\limits_{i = 1}^n \left( {y_{obs,i} - y_{pred,i}} \right)^2}$$

where $\bar Y = \frac{1}{n}\mathop {\sum }\limits_{i = 1}^n y_{obs,i}$ is the mean of the *n* observed numbers of survivors $y_{obs,i}$ for i = 1,…,*n*. Numbers $y_{pred,i}$ correspond to the median of the predicted numbers of survivors at each time-point. A third criterion for the model accuracy at the end of the experiments was developed based on survival probabilities. The probability to survive an exposure profile from the beginning to the end of an experiment is given as ratio between the number of surviving (Y_obs,tend_) and the number of initial individuals (Y_init_) in a test, hence the difference between the observed and modelled survival probabilities, or in other words the survival probability prediction error (SPPE) is given as

2 $$\begin{array}{lcc}{\mathrm {SPPE}} = \frac{{Y_{obs,tend}}}{{Y_{init}}} - \frac{{Y_{modelled,tend}}}{{Y_{init}}} \ast 100 \\ = \frac{{Y_{obs,tend} - Y_{modelled,tend}}}{{Y_{init}}} \ast 100 \end{array}$$

The SPPE is suggested as indicator of model accuracy considering survival probabilities only at the end of a tested exposure profile. The SPPE indicator is negative (between 0 and −100%) for an underestimation of effects, and positive (between 0 and 100%) for an overestimation of effects. An SPPE value of 0% means an exact prediction of the observed survival probability at the end of the experiment. All indicator calculations are given in ESM6 (ESM6_indicator-calculations.ods).

## Results

### Model calibration

**Fig. 1:**
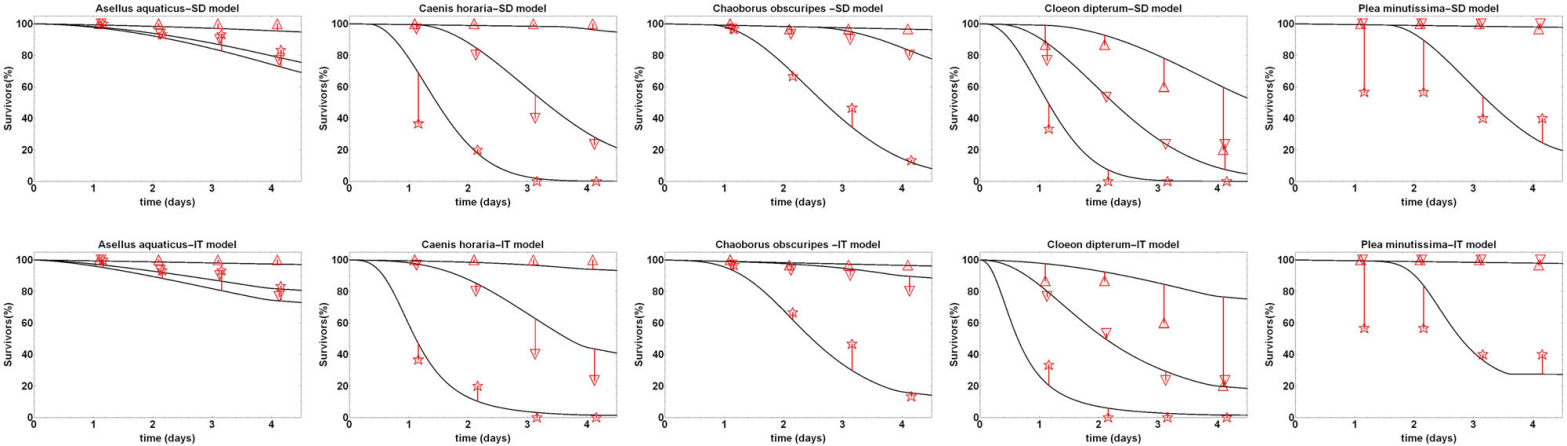
Calibration fits of the GUTS-RED-SD and GUTS-RED-IT models (see Material and Methods) to survival data of experiment MS_C. Species names are given in the title of the single plots. Symbols show observed numbers of living organisms for the three highest treatment levels (exp. MS_C). Fits for all treatment levels and height of exposure concentrations can be found in ESM3. The lines show the fitted SD and IT models

The models were calibrated to the observed survival counts from the acute tests in experiment MS_C1 (Fig. 1). Visual inspection of the calibrated SIC-SD and SIC-IT models with the observed data indicated a good visual match between modelled and observed survival under increasing treatment levels for 4 out of 5 species. For the backswimmer larvae *P. minutissima*, only the highest treatment level resulted in a visible effect on the observed survival of the tested organisms, and the number of living individuals changed after day 1 only in 1 of 3 replicates, which prevented a better fit of the GUTS models. In general, the SD model had lower negative log likelihood values than the IT model, indicating a better fit of the SD model to the data set (Table S2).

**Fig. 2:**
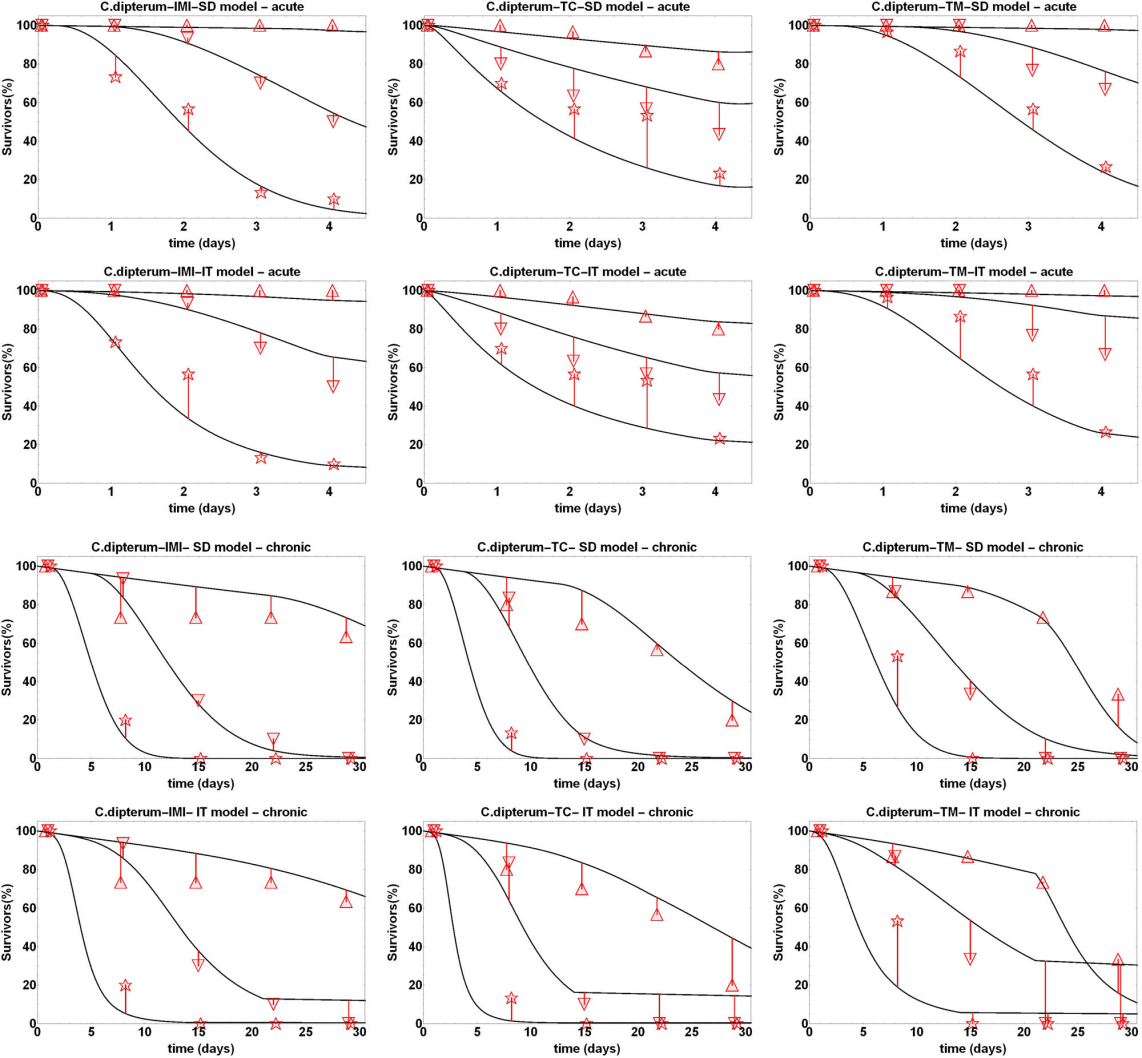
Calibration fits of the GUTS-RED-SD and GUTS-RED-IT models (see Material and Methods) to survival data of experiment MC_C1 (acute experiments, top rows) and MC_C2 (chronic experiment, bottom rows). Species and compound names are given in the title of the single plots. Symbols show observed numbers of living organisms for the three highest treatment levels. Fits for all treatment levels and height of exposure concentrations can be found in ESM3. The lines show the fitted SD and IT models

For model calibration for imidacloprid, thiacloprid and thiamethoxam on *C. dipterum*, observed survival counts from experiment MC_C1 were used. Differently than for the other data sets, nominal concentrations were used for the calibration because concentrations have been measured only in the lowest and highest treatment levels (Van den Brink et al. 2016). Visual inspection of the model fits indicated that survival patterns in the data were fitted well (Fig. 2) with SD and IT model performing nearly equal as indicated by very similar log-likelihood values (Table S2).

A second parameter set for the effect of imidacloprid, thiacloprid and thiamethoxam exposure on the survival of *C. dipterum* was obtained from fitting the SIC-SD and SIC-IT models to observed survival counts from a chronic test (experiment MC_C2; Fig. 2). The 1 μg/L treatment of thiamethoxam in the MC_C2 data set was wrongly dosed on day 21, when the 10-fold of the intended dose was applied. This resulted in a clear increase in mortality in the last week of the experiment, which could be fitted well by both models. The SIC-SD model was showing similar LL values compared to the SIC-IT model (Table S2).

As a result of the calibration routines, one parameter set per SIC-SD and SIC-IT model was obtained for each of the five macroinvertebrate species and imidacloprid and two parameter sets per SIC-SD and SIC-IT model were obtained for *C. dipterum* and three neonicotinoids, one based on acute and one based on chronic test results. Confidence limits for all model parameters were approximated using the likelihood profiling method (Table S2).

### Multiple species validation experiments and model predictions

Species with a wide range of sensitivities were selected for the validation experiments MS_V: from the very sensitive *C. horaria* and *C. dipterum* over the intermediate sensitive *P. minutissima* and *A. aquaticus* up to the relative insensitive *C. obscuripes* (Van den Brink et al. 2016).

**Fig. 3:**
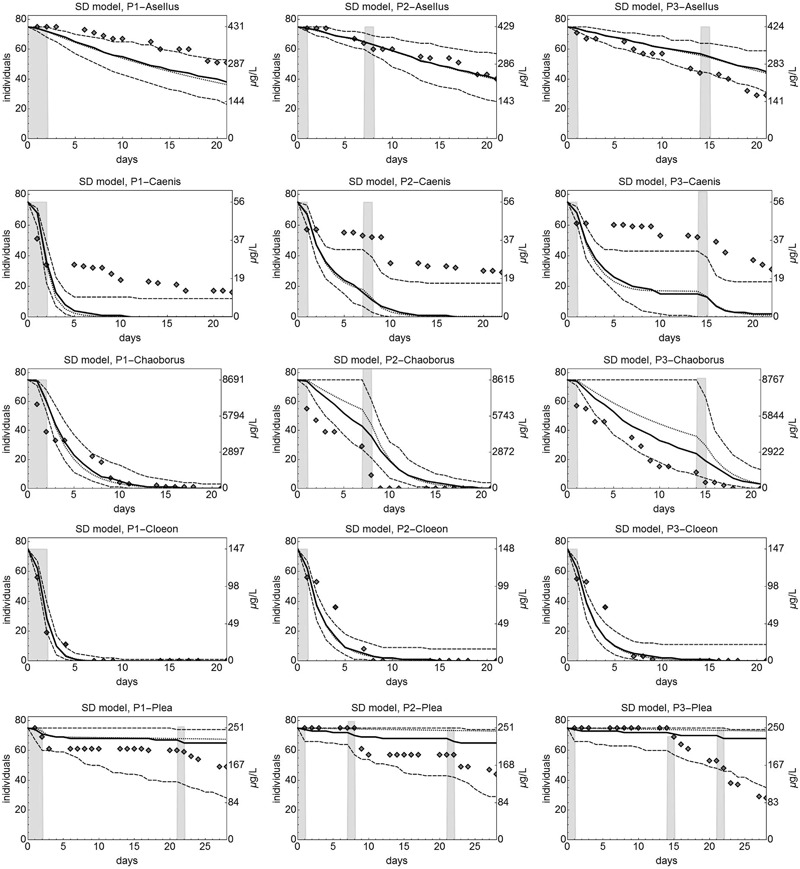
Observed and predicted survival of individuals of 5 macroinvertebrate species under 3 pulsed exposure profiles for imidacloprid, predicted by the stochastic death (SD) model calibrated to data from on acute testing (exp. MS_C). Diamonds indicate observed numbers of living organisms (exp. MS_V). The dashed lines show the 5 and 95 percentile of 10.000 probabilistic simulations of the time course of survival (see ESM2.2.4), the solid line is the respective median. Black dotted lines show the predicted deterministic rate of survivors. Grey bars show the exposure levels over time, measured concentrations as indicated in the axes labels on the right hand side of the single panels. For *C.dipterum*, in the P2 and P3 teratment no second pulse was applied becauyse almost all individuals were already dead

Observed survival for *A. aquaticus* in experiment MS_V showed no recovery between pulses (Fig. 3). Whereas in treatment P1 51 individuals survived until day 28, this number was 40 for P2 and 29 in P3. Visual inspection of the SD model predictions indicated a reasonable match with the observed survival pattern for most treatments, and a very good one for the P2 treatment. However, the model predictions did not match the pattern of increased effects with increasing interval between the exposure pulses. In contrast, a small recovery effect was predicted by the model, as the predicted median number of living individuals at day 21 increased from 38 for P1 over 40 individuals for P2 to 45 individuals for P3. The IT model predictions for *A. aquaticus* matched the data less than the predictions of the SD model (Fig. S2). The observed survival was in general overestimated by the IT model. These observations are also reflected by the corresponding indicator values (Table 2): The IT model predictions resulted in larger LL values and also in larger NRMSE and MPE values. The SD model resulted in survival probability prediction errors (SPPE) closer to zero, which indicates a good match of the observed and predicted survival probabilities at the end of the validation experiments.

**Table 2:** Indicator values calculated for the quality of predictions for different validation data sets. Definition of the indicator values in Material and Methods, Section 2.2.3)

			SD model				IT model			
Data set	Species/compound		LL	MPE (%)	NRMSE	SPPE (%)	LL	MPE (%)	NRMSE	SPPE (%)
Multiple species (MS_V) predicted based on MS_C1	*A. aquaticus,* imidacloprid	**P1**	−472.635	20%	**16.7%**	17%	−533.308	7%	**9.8%**	−17%
		**P2**		3%	**4.3%**	0%		14%	**20.2%**	−31%
		**P3**		15%	**18.5%**	−21%		25%	**37.9%**	−45%
	*C.horaria,* imidacloprid	**P1**	−852.624	n.d.	74.2%	21%	−598.646	22%	**22.7%**	−7%
		**P2**		n.d.	69.1%	39%		43%	**29.3%**	11%
		**P3**		504%	63.6%	39%		30%	**20.6%**	15%
	*C.obscuripes,* imidacloprid	**P1**	−430.397	n.d.	**42.9%**	0%	−891.046	71%	239.8%	−76%
		**P2**		n.d.	85.5%	0%		75%	258.2%	−75%
		**P3**		49%	**49.4%**	−4%		65%	177.2%	−75%
	*C.dipterum,* imidacloprid	**P1**	−289.423	n.d.	**30.1%**	0%	−917.499	75%	136.8%	−27%
		**P2**		n.d.	**42.3%**	0%		72%	215.1%	−60%
		**P3**		n.d.	**42.1%**	0%		72%	217.7%	−61%
	*P.minutissima,* imidacloprid	**P1**	−663.204	11%	**13.6%**	−21%	−695.424	16%	**20.6%**	−29%
		**P2**		14%	**17.7%**	−28%		17%	**25.8%**	−39%
		**P3**		16%	**27.5%**	−53%		17%	**31.8%**	−60%
Multiple compounds (MC_V) predicted based on MC_C1 (acute)	*C.dipterum*, imidacloprid	**P1**	−28.818	19%	**22.3%**	−23%	−29.811	29%	**42.5%**	−40%
		**P2**		17%	**19.0%**	−20%		24%	**35.8%**	−40%
	*C.dipterum*, thiacloprid	**P1**	−40.701	59%	127.5%	−60%	−41.103	59%	141.2%	−67%
		**P2**		31%	**44.1%**	−30%		33%	52.0%	−37%
	*C.dipterum*, thiametoxam	**P1**	−26.990	48%	77.0%	−53%	−29.441	52%	114.5%	−77%
		**P2**		52%	99.4%	−60%		54%	125.4%	−77%
Multiple compounds (MC_V) predicted based on MC_C2 (chronic)	*C.dipterum*, imidacloprid	**P1**	−170.030	n.d.	72.7%	27%	−174.501	n.d.	78.1%	27%
		**P2**		n.d.	76.2%	30%		n.d.	76.7%	30%
	*C.dipterum*, thiacloprid	**P1**	−167.871	n.d.	54.2%	0%	−182.411	143%	77.8%	−3%
		**P2**		n.d.	65.1%	33%		470%	63.5%	30%
	*C.dipterum*, thiametoxam	**P1**	−171.581	n.d.	**46.4%**	0%	−186.249	85%	54.7%	−13%
		**P2**		n.d.	**37.0%**	0%		45%	51.5%	−13%

The bold values are larger than a suggested cut-off value of 50%

n.d. not determined because modelled survival fell to zero

For *C. horaria*, the observed survival showed the expected pattern: for longer no-exposure intervals the effects decreased presumably because of depuration or repair between pulses. The number of living individuals at the last day increased from 16 for P1 to 29 and 31 individuals for P2 and P3, respectively (Fig. 3). The difference between P2 and P3 was not very pronounced, hence it appears that 6 days between pulses was long enough for *C. horaria* individuals to establish the maximum level of recovery. The SD model predictions of the number of living individuals underestimated the observed survival for all three profiles (Fig. 3), but matched the observed pattern in the survival observation with almost constant survivor numbers between the pulses. The IT model (Fig. S2) predicted the survival over time under all three tested profiles for *C. horaria* better than the SD model, as reflected by smaller NRMSE values (Table 2). Also, predictions of the final effects were better for the IT model, as indicated by SPPE value closer to zero. Notably, calculation of the MPE indicators for the SD model was not possible because modelled survival fell down to zero.

For *C. obscuripes*, the experimental treatments resulted in rapidly decreasing survival over time (Fig. 3). Only low mortality was observed in the control (< 10%), hence the decrease of survivors was a clear toxicant effect. For all three tested profiles, all individuals were dead at the end of the experiment. These patterns are qualitatively well matched by the SD model predictions, especially for the P1 treatment, while for the P2 and P3 treatments the predictions slightly overestimated the observed survivor numbers. Remarkably, the confidence limits of the predicted survival included full survival between the pulses in P2 and P3, indicating a relative high uncertainty. The IT model failed to predict the survival patterns observed for *C. obscuripes*, it clearly overestimated the observed survival (Fig. S2).

For *C. dipterum*, all three treatments resulted in a rapid decline of the number survivors, for P1 somewhat faster than for P2 and P3 (Fig. 3). No second pulses were applied in P2 and P3, because at the respective time points almost no individuals were alive. Differentiation between the effects of the treatments on the observed numbers of survivors was hardly possible. Apparently, the chosen pulse concentrations caused stronger effects than anticipated. Hence, the differences in survival over time were not pronounced between the treatments. This pattern was qualitatively and quantitatively matched by the SD model predictions, whereas the IT model failed to predict the high mortality observed in all three treatments (Fig. 3 and Fig. S2). Consequently, the log-likelihood value for the SD model is clearly smaller than the one for the IT model, smaller NRMSE values indicate a better match of the SD model to the observed survival over time as compared to the IT model, and SPPE values equal to or close to zero reflect the good matching of the final survival by the SD model predictions (Table 2).

For *P. minutissima*, the later the second application of imidacloprid was added, the lower was the survival at the end of the experiment (Fig. 3), similar to the *A. aquaticus* data. A third pulse was added in all three treatments at day 21, resulting in additional mortality. The P1 treatment resulted finally in 49 living individuals, whereas in the P2 treatment 44 and in P3 28 individuals survived at day 28. None of these patterns were, however, captured by the predictions of the SD and the IT model (Fig. 3 and S2, respectively). Both models overestimated the observed survival. At least, most observed survivor numbers of *P. minutissima* were within the uncertainty range of the probabilistic model predictions for the SD model, indicating that the observed survival over time is within the biological variability as captured by the SD model.

**Fig. 4:**
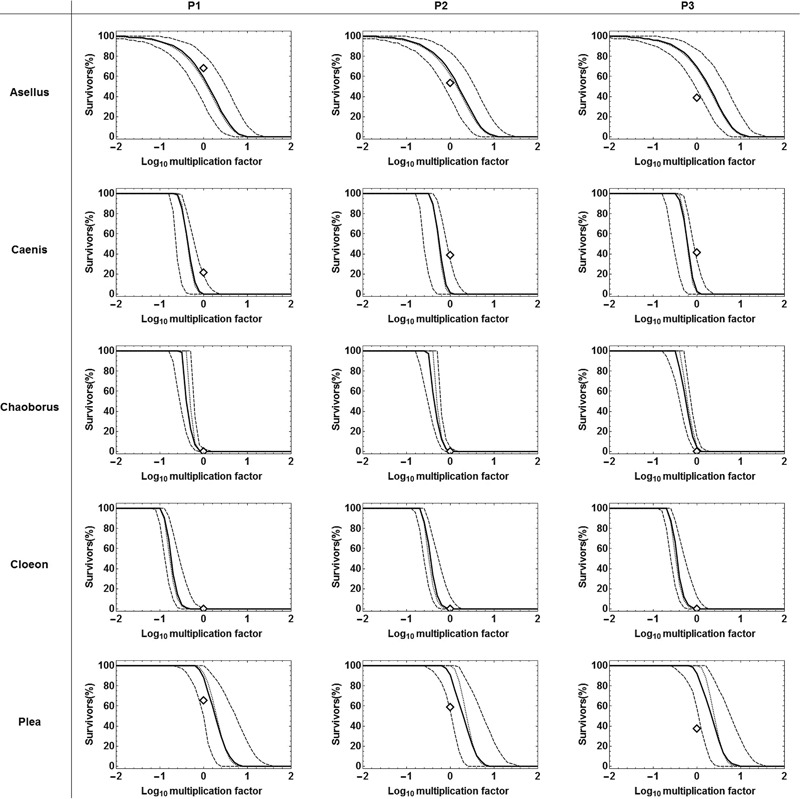
Predictions of exposure-profile specific dose-response curves for the 3 tested pulsed exposure regimes for imidacloprid, based on calibration to results from acute testing (exp. MS_C1) using the SD model. Diamonds indicate the observed number of living organisms (exp. MS_V) at the end of a specific experiment. The dashed lines shown the 5 and 95 percentile of 10,000 probabilistic simulations of the time course of survival (ref. section ESM2.2.4.3) per multiplication factor, the solid line is the respective median. Black dotted lines show the deterministic rate of survivors

Exposure profile specific dose-response curves were calculated based on the calibrated SIC-SD models (Fig. 4). For *A. aquaticus*, the median dose-response curve was indeed lower than the observed survival for the P1 profile, whereas for the P2 profile the predicted number of survivors was slightly higher than the predicted, even though still within the uncertainty region between the 5th and the 95th percentiles of the probabilistic predictions. For the P3 profile, the predicted survivor number was clearly higher than the observed. For *C. horaria*, all SD model predictions are indicating lower survival than it was observed for the three tested profiles. Observations for *C. obscuripes* and *C. dipterum* are similar to each other: for both species no individual survived any of the three tested exposure profiles, what was correctly predicted by the SD model. For *P. minutissima*, the SD model based predictions overestimated the survival for all 3 tested exposure profiled, even if the observed survival is within or close to the uncertainty region between the 5th and 95th percentile of the probabilistic predictions.

### Multiple compounds validation experiments and model predictions

**Fig. 5:**
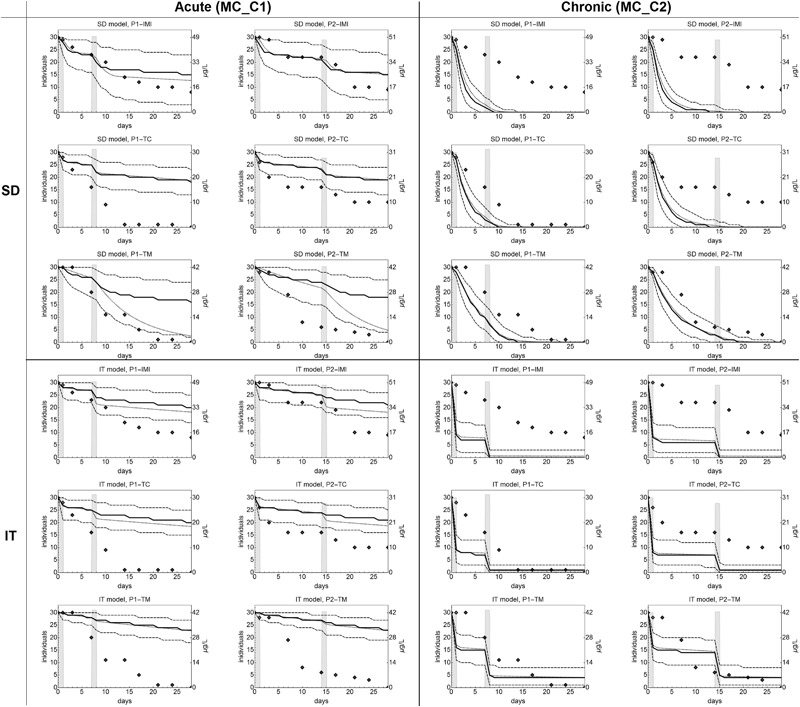
Observed and predicted survival in experiment MC_V. The single plots show survival of *C. dipterum* under exposure to imidacloprid (IMI), thiacloprid (TC), thiametoxam (TM)) with the SD model (3 top rows) and the IT model (3 bottom rows), model calibration based on data from acute (MC_C1, left columns) or chronic experiments (MC_C2, right columns). Diamonds indicate observed numbers of living organisms (exp. MC_V). The dashed lines shown the 5 and 95 percentile of 10.000 probabilistic simulations of the time course of survival (see ref. ESM2.2.4), the solid line is the respective median. Black dotted lines show the deterministic rate of survivors. Grey bars show the exposure levels over time, measured concentrations as indicated in the axes labels on the right hand side of the single panels

For imidacloprid, the treatment P1 resulted in continuously decreasing survivors of *C. dipterum* over time (Fig. 5). In treatment P2, the decrease halted between day 7 and day 14, and continued after the second pulse at day 14. Apparently, the timing of the second pulse did not matter much for the final effect, since for the P1 profile 8 and for the P2 profile 9 individuals of initially 30 survived.

Model predictions were calculated based on calibration on data from both acute (MC_C1) and chronic (MC_C2) experiments. The SD model predictions for imidacloprid based on acute experiments matched the observed pattern of survivors visually well up to the second pulse in both the P1 and the P2 treatment (Fig. 5, left top corner). However, the decrease of survivors after the respective second pulses was quantitatively not matched by the predictions. Final predicted numbers of survivors overestimated survival, resulting in SPPE values of −23% and −20% (Table 2). When the SD model was calibrated on chronic experiments (MC_C2) instead, the predictions strongly overestimated mortality. For both exposure profiles, the modelled numbers of surviving organisms fell to zero before day 14, apparently independent from the respective second pulses. The IT model predictions showed similar patterns, and clear differences between the P1 and P2 treatments for the chronic calibration, but also overestimated the observed effects strongly (Fig. 5).

The patterns of observed mortality was different between the P1 and P2 exposure profiles of thiacloprid. Whereas in P1 the number of survivors steadily decreased reaching zero at day 14, in the P2 treatment the number of surviving individuals was constant at days 7, 10, and 14 and decreased after the second pulse to finally 10 surviving individuals (Fig. 5). Initial mortality and additional effects of the second pulses in both the P1 and P2 treatments are visible in both the SD and IT model predictions for calibration on acute data. However, the final effects on survival were underestimated leading to SPPE values of −30 % and −60% for the SD model and the P1 and the P2 treatments, respectively, and −37% and −67% for the IT model predictions (Table 2). The SD model predictions based on chronic tests predicted continuously decreasing survival already after the first thiacloprid pulse and overestimated the final effects on the *C. dipterum* survival, while the IT model calibrated on chronic tests also over-predicted the final effects but matched the pattern in the observed survival data a bit better. Smallest NRMSE values are indicated for the SD model predictions based on acute data.

For thiamethoxam, both treatments resulted in complete mortality on the last experimental day. The survival over time differed only slightly between day 10 and day 18 for the P1 and P2 treatments (Fig. 5). Predictions of the number of living individuals as calculated with the SD model calibrated on acute test data showed a steadily decreasing number of survivors for both treatments. Remarkably, the deterministic rate of survival predicted by using the optimal parameter set was very different from the median of the probabilistic predictions for this case. This is exceptional, and probably relates to a skewed confidence region. The deterministic prediction is only slightly higher than the observed survival, whereas the median survival is far too high compared to the experimental observations. The latter is also the case for the IT model predictions. The survival as predicted by the SD model based on chronic tests matches the observed data well, and resulted in smallest NRMSE values and SPPE values of 0% for both treatments, whereas the IT model predictions showed too high survival at the end of the experiments.

Comparing the predictions of the number of surviving individuals with the models using the acute (MC_C1) or the chronic (MC_C2) datasets for calibration, it is obvious that the acute-based model calibration results in an under prediction of observed effects, or in other words, a lower accuracy as compared to the chronic-based model. There is a good visual match between the acute-based SD model predictions and the pattern of observed surviving individuals for imidacloprid, whereas for thiamethoxam and slightly less for thiacloprid the observed effects on survival were matched better by the SD model calibrated to chronic test results (Fig. 5).

## Discussion

### Evaluation of model predictions for multiple species

The comparison of modelled and observed survival of the tested macroinvertebrates under time-variable exposure profiles allows for an evaluation of the prediction quality. For the multiple species data set, the observed patterns of survival were qualitatively matched by predictions of the SD model for 4 of 5 tested species. In this context, qualitative matching means that the trend in the observed data, e.g., time points of decrease, periods with constant survival or continuously decreasing survival are observable in the simulations, but that the simulations do not match the absolute values of observed numbers. Only for *P. minutissima*, the median of the probabilistic model predictions showed nearly no response to exposure peaks, unlike which was observed, while the lower uncertainty limit of the model predictions included most of the observed numbers of survivors. For the other species, the patterns or trends in the data, e.g., the decrease of surviving individuals in response to a next pulse, were reproduced by the model predictions, even though the absolute numbers of survivors were not matched in all cases, for example for *C.horaria* (Fig. 3).

SD model predictions for all pulsed scenarios for *A. aquaticus, C. dipterum* and with limitations also *C. obscuripes* appear of acceptable quality, as indicated by lower LL values in comparison to the LL values of the IT model. MPE values could not be calculated in many cases, because the modelled survival reached zero at the end of exposure (Table 2). NRMSE values for the three mentioned species are lower than 50%, hence indicating a reasonable match between predicted and observed survival over time. Also the SPPE values are close to zero or large, with exception of P3 for *A. aquaticus*, hence indicating a good-to-conservative match of the final number of survivors. *P. minutissima* and *C. horaria* showed survival patterns in the validation experiments similar to what Ashauer and colleagues recently classified to be typical for individual tolerance and slow toxicodynamic recovery: fast reduction of the survivor numbers to a lower, but then constant level (Ashauer et al. 2015). Consequently, the observed survival for *C. horaria* was predicted better by the IT model, what is expressed in lower LL and NRMSE values. The latter values are below 30%, hence showing that IT model predictions were quite good, which is also qualitatively confirmed (Fig. S2). In contrast to the described pattern of individual tolerance, the response of *A. aquaticus, C. obscuripes* and *C. dipterum* indicated clear patterns of stochastic death under slow toxicodynamic recovery, because a pulse event is followed by a constant decrease in abundance. Survival data for these three species were matched better by the SD model predictions. For *P. minutissima*, predictions of both the SD and the IT model did not match the survival as observed in the validation experiments, most probably due to the incomplete dose-response relationship in the calibration experiments. The case of the mismatch of the *P. minutissima* predictions, and to a lesser extend also for *A. aquaticus*, underlines the importance of appropriate calibration data, which should ideally show clear effects in several tested treatment levels. Interestingly, NRMSE values are showing quite small values below 30% for the SD model and below 35% for the IT model, despite a qualitative mismatch between the model predictions and the observed dynamics. This finding highlights that for the validation of GUTS model predictions, a combination of qualitative and quantitative indicators should be evaluated.

It appears beneficial that model predictions were tested with validations data sets for a number of different species, as it has already been suggested by Nyman et al. (2012), because obviously different species show different responses to time-variable exposure profiles of the same chemical, hence from the quality of model predictions for a single species-compound combination it is not possible to conclude about the quality of model predictions for other species-compound combinations. For 4 of 5 tested species, however, a good match between model predictions and observed data could be identified by a combination of qualitative and quantitative assessments. Only for *P. minutissima* predictions were clearly mismatching the observed survival, with the mentioned ‘incomplete’ calibration data as possible reason. An additional possible reason for prediction mismatches for *P. minutissima* could be that there were 6 weeks between calibration and validation experiments. For *P. minutissima*, the acute tests (exp. MS_C1) were performed in the beginning of October 2014, while the validation tests (exp. MS_V) were performed in the end of November (Table 1). It might well be that within these 6 weeks, where environmental temperatures and light conditions have changed substantially, also the physiology of some individuals changed and with that the sensitivity towards imidacloprid. Such changes can be more pronounced in some species than in others. Van den Brink et al. (2016) reported LC_50_ values (4d) for *P. minutissima* that differed by a factor of 7.8 between tests in May/June and October, supporting that for *P. minutissima* physiological changes can play an important role on longer time scales.

### Quality of model predictions for multiple compounds

From mode of action considerations, it is known that receptor binding affinity and specificity to the nicotinic acetylcholine receptor (nAChR) appears equivalent among different neonicotinoids (Zhang et al. 2000). Therefore it can be expected that different neonicotinoid compounds show similar effects in the same species, on the other hand side, neonicotinoid molecules differ in their configuration. Imidacloprid and thiamethoxam are reported to contain an electronegative nitro-substituted heterocyclic group, whereas thiacloprid contains a cyano- substituted heterocyclic group that confers a higher detoxification potential (Morrissey et al. 2015; Manjon et al. 2018). Van den Brink et al. (2016) and Morrisey et al. (2015) reported in accordance that imidacloprid and thiamethoxam exhibit similar toxicity to *C. dipterum* and insects in general, while thiacloprid shows a higher toxicity. Consequently, the pulsed tests were adapted according to the differences in toxicity by targeting LC_30_ concentrations for the height of the pulses (Table S1).

For imidacloprid, the timing of the second pulses changed the mortality pattern over time, but not the final mortality (Fig. 5). This might be explained by the irreversibility of the imidacloprid effect, leading to long recovery times being necessary between pulses to make them toxicologically independent. In contrast, for thiacloprid the time point of the second pulse had an influence on the final effect. One possible explanation for this observation would be that the individuals in the P2 treatment recovered from the first pulse, while in the P1 treatment the time was not sufficient for detoxification. Possibly a higher detoxification potential of thiacloprid than for imidacloprid and thiamethoxam as given by the differences in the respective molecular configuration could play a role for aquatic arthropods, as it was reported for honey bees as well (Iwasa et al. 2004; Manjon et al. 2018). Observations for thiamethoxam can be interpreted similar to the imidacloprid effect showing irreversibility of damage within the tested time period.

The accuracy of predictions was different for models being calibrated on acute or on chronic data. For imidacloprid, the SD model predictions based on acute data showed the lowest NRMSE values, indicating an acceptable fit of the survival over time, while for thimethoxam the SD model based on chronic data showed better NRMSE values. For thiacloprid, neither the SD nor the IT model predictions gave NRMSE values below 50% for both tested exposure profiles, hence scientifically no really satisfying prediction was seen. Nevertheless, model predictions were at least conservative using the SD model based on chronic testing.

In general, the exposure duration in the calibration experiments has a strong influence on the predicted effect magnitude. Model predictions based on acute test results in generally underestimated the observed effects of pulsed exposure on survival, and predictions based on chronic experiments were matching the observed survival in case of thiamethoxam or overestimated the observed effects for imidacloprid and thiacloprid (Fig. 5). This result could be explained by earlier findings about the influence of exposure time on effect (Tennekes 2010; Tennekes and Sánchez-Bayo 2013), which report toxic effects that are increased with time of exposure, i.e., time-cumulative effects for compounds that exhibit specific and irreversible binding to the target enzyme. Neonicotinoids are suspected to belong to this class of compounds, hence in theory stronger effects can be expected for longer exposure times. When using survival data under long-term exposure for model calibration, predicted effects on survival can consequently be expected to be higher than when observed in short-term pulsed exposure.

### Validation status of the tested TKTD models

Toxicokinetic-toxicodynamic models can play an important role to overcome the current limitation in environmental risk assessment of not having the same time pattern of exposure in ecotoxicological experiments and real-world field exposure situations (Ashauer and Brown 2013; Ashauer and Escher 2010; Ducrot et al. 2016). TKTD models can in this context be used for very useful calculations, such as to screen large numbers of exposure scenarios, or as it has been shown by Ashauer et al. (2013) to calculate the margins of safety for risk assessment. Necessary for the acceptance of TKTD modelling results in science, but especially for acceptance for regulatory risk assessment is to increase the number of examples of validated application cases. With this study, we aimed at increasing the number of validation case studies for a relevant compound class. We reported here about the first study on calibration and validation of GUTS models for neonicotinoid compounds.

First of all, the MPE indicator appears not useful for the evaluation of the quantitative match between predicted and observed survival over time, since in many cases values could not be calculated. SPPE and NRMSE values could always be calculated and appear more suitable for model validation.

Scientifically, results are in parts not satisfying, since simulation results are in some cases massively diverging from the observed values, as reflected by the ‘visual fit’, and high SPPE and NMRSE values. Most predictions with a bad fit are seen for calibration on acute data and the predictions of survival under pulsed exposure to TC or TM. Predictions of survival under exposure to IMI matched the observed data relatively well (Figs. 3 and 5). For 4 out of 5 the tested species in the multiple species data predictions for IMI, model predictions for risk assessment appear acceptable, based on the visual fit and the SPPE and NRMSE indicators, which show in most combinations of compounds, pulsed scenarios and species values below 50%. The results indicate that using the more conservative of SD and IT model predictions calibrated to acute test results, GUTS model predictions have a good chance of matching observed survival for imidacloprid. Nevertheless, for thiacloprid and thiamethoxam, predictions based on acute exposure were under-predicting the effects of pulsed exposure on the survival. Only when calibrating the SD and IT models based on data from chronic exposure, acceptable predictions were obtained. In conclusion, it appears that for compounds which are known to have the potential to increase in toxicity over exposure time, for example due to irreversible binding to target enzymes, calibration and validation of TKTD models for survival needs to be performed with special attention to the duration of exposure in the calibration experiments. In this context, it appears useful that most often not only acute, but also chronic datasets are available in a standard risk assessment procedure. Both acute and chronic data should be checked and used for GUTS model calibration.

In addition to the assessment of the matching of the survival over time, dose-response figures can provide useful aspects to the evaluation (Fig. 4, S3). Such dose-response view reflects the use of toxicity information in the environmental risk assessment of chemicals, with focus on the effect at the end of a specific exposure pattern. The risk-assessment relevant question for the evaluation of those dose-response relationships is whether the TKTD model based predictions are indicative of possible toxic effects of a specific tested exposure profile at the end of the test period. This is the case when the median dose-response curve is lower than or equal to the observed survival at the end of the experiments (Fig. 4; S3). Dose-response plots indicate not only whether the observed survival at the end of a tested exposure profile was matched, but they capture additional information about model predictions for experimentally untested exposure profiles and can be combined with uncertainty analyses, so that exposure profile specific toxicity thresholds could be derived from such curves including confidence limits, e.g., the multiplication factor necessary to reach 50% effect. Predicted dose-response curves allow for a check of the accuracy of the location of the mid-point of the predicted dose-response curve in comparison. It would provide more useful insight, when more than one, e.g., at least three different pulse concentrations per profile would be tested in future validation experiments, because then not only the mid-point but also the slope of dose-response view could be evaluated. Nevertheless, the data set used for this study allows to identify at least the accuracy of the mid-point of the predicted dose-response curves or at least conservative for 4 of 5 tested species.

The multiple species and multiple compound experiments contained both datasets for imidacloprid and *C. dipterum*, which would provide the opportunity for additional validation. In this study, this opportunity was not taken because the modelling was already very comprehensive.

Comparing our validation results to other studies in the literature shows that for most of the validation cases also good or even better matches between observed and predicted survival were found (Ashauer et al. 2007a, b). For the studies of Ashauer et al. (2007a, b), however, toxicokinetics was measured separately, and model calibration was done on pulsed exposure and not on standard acute test results. Our results corroborate earlier findings that standard toxicity data sets could be used for the calibration of GUTS models using the scaled internal concentrations as dose metric (Nyman et al. 2012).

### Usability of the models for risk assessment

Accepting the limitation that validation experiments should have included more concentration levels per tested profile to provide the means to test also the slope of predicted dose-response curves, SD model parameterisation for imidacloprid and *A. aquaticus, C. obscuripes* and *C. dipterum* results in prediction qualities that appear acceptable for the use of in risk assessment, and the same holds for imidacloprid and *C. horaria* and the IT model. For thiacloprid and thiamethoxam, predictions with the SD model calibrated to data from chronic experiments resulted in scientifically not completely convincing, but sufficiently conservative predictions to enable the use of such predictions in regulatory risk assessment. A complete dose-response pattern in the experiments used for model calibration would probably have allowed more accurate predictions also for *P. minutissima*.

The application of TKTD modelling for environmental risk assessment appears possible for most of the presented species-compound combinations, when both SD and IT models are evaluated and additional care is taken of the use of information about toxicity under long-term exposure. Predictions of the mortality risk for time-variable exposure scenarios can be calculated without the need of conducting extra experiments to measure the toxicokinetics of compounds.

## Electronic supplementary material

Measured Exposure

Additional material and methods

additional figures

additional tables

raw survival data

indicator calculations
