# Supplementary material for: Calibration and validation of toxicokinetic-toxicodynamic models for three neonicotinoids and some aquatic macroinvertebrates
Source: Ecotoxicology. 2018 May 1;27(7):992–1007. doi: 10.1007/s10646-018-1940-6 (PMC6132984; doi:10.1007/s10646-018-1940-6)
Supplement: Supplementary file 2 — Additional material and methods [file 10646_2018_1940_MOESM2_ESM.docx]

**Supporting information for the manuscript**

**Calibration and validation of toxicokinetic-toxicodynamic models for three neonicotinoids and some aquatic macroinvertebrates**

Andreas Focks^1^*, Dick Belgers^1^, Marie-Claire Boerwinkel^1^, Laura Buijse^1^, Ivo Roessink^1^, Paul J. Van den Brink^1,2^

^1^Alterra, Wageningen University and Research centre, P.O. Box 47, 6700 AA Wageningen, The Netherlands

^2^Department of Aquatic Ecology and Water Quality Management, Wageningen University, Wageningen University and Research centre, P.O. Box 47, 6700 AA Wageningen, The Netherlands

* Corresponding author: andreas.focks@wur.nl

Table of Contents

[1. Additonal infromation about experiments MC_C2 and MC_V 2](#_Toc510179356)

[2. Addtional modelling information 2](#_Toc510179357)

[2.1. The GUTS models 2](#_Toc510179358)

[2.2. Calibration of model parameters 3](#_Toc510179359)

[2.3. Numerical estimation of parameter confidence intervals 3](#_Toc510179360)

[2.4. Model predictions and calculation of model performance statistics 4](#_Toc510179361)

[2.4.1. Stochasticity of survival and probabilistic modelling of survival over time 4](#_Toc510179362)

[2.4.2. Including parametric uncertainty in forecasts 4](#_Toc510179363)

[2.4.3. Dose response curves for pulsed exposures 5](#_Toc510179364)

[2.4.4. Calculation of model performance statistics 5](#_Toc510179365)

# Additonal information about experiments MC_C2 and MC_V

In a chronic test (experiment **MC_C2)**, the survival of *Cloeon dipterum* was tested for six treatment levels in triplicates and nine controls. In experiment **MC_V**, under each two different pulsed exposure regimes to imidacloprid, thiacloprid and thiamethoxam was investigated in each three replicates. In additional controls, the survival of C. dipterum without exposure was observed in 9 replicates. In both experiments, each test system contained 10 *C. dipterum* individuals. Test animal collection was according to Van Den Brink et al. (2016). Immediately after collection of the animals (3 days before dosing) they were transported to the laboratory and preserved in aerated aquaria (50% groundwater and 50% surface water) at least for three days to acclimatize the animals to indoor laboratory condition. During acclimatization and test periods, animals were fed on species specific food, i.e. a combination of conditioned organic matter (four small rounds (ø 14 mm) of populous leaves), periphytic algae and *Elodea nuttallii* (top shoot, 5 cm). Deep groundwater from a well at the Sinderhoeve test facility, was aerated for at least 24 hours before animals were added to this water. Test water was collected 5 days before dosing. After collecting it was stored in a climate room, 18°C ± 2. Temperature, pH, dissolved oxygen (DO) and electrical conductivity (EC) were measured weekly at approximately mid depth in the experimental systems. More details about experimental conditions are given in the SI.

The test systems were placed in a climate room at 18°C ± 2 °C. A photoperiod of 16:8 hours dark-light was applied. Test systems were spiked with the required volume from appropriate stock solutions. New systems were set up every 7 days before refreshment, by introducing organic matter, algal diatoms and *E. nuttallii* into jars containing 1000 ml test water. The water level in the jars was 12.5 cm. The jars were cultured under daylight lamps (light intensity 180-200 uS/Cm) for one week prior to refreshment. Effects were scored as mortality when no response of any kind was observed over a time period of 3-5 seconds after tactile stimulation or prodding. In addition, care was taken that any emerging individual did not escape the test vessels by placing a gauze mesh over the opening of the jar. Emerged and dead individuals were removed from the test. Living individuals were counted on days 7, 14, 21, 28.

# Addtional modelling information

The model equations have been documented earlier (Jager et al, 2011) and are given here for comprehensiveness. The model parameters were calibrated using the calibration data sets and then used for predictions of survival given the measured exposure profiles in the validation experiments.

Model calibration was done using survival counts and concentration measurements from the acute or chronic tests for each replicate, if not stated differently, to account for differences in the exposure levels between replicates. Model validation was done using survival counts from the pulsed experiments combining the replicates per treatment, and respective concentration measurements were averaged over the replicates. Differences between the single pulse concentrations were larger than between the replicates.

The numbers of individuals per treatment in the validation experiments was small (<100), so model predictions were calculated under consideration of the stochasticity of survival in a small cohort of individuals (see Albert et al., 2012). For model validation, the median of model predictions was compared to observed survival, and Chi^2^-statistics were calculated as a measure of the goodness of fit.

## The GUTS models

***Dose metric – scaled internal concentration***

The scaled internal concentration *C*_i_*(t) was used as a dose metric (Jager et al., 2011). The time course of the scaled internal concentration is determined by the dominant rate constant *k_D_* (time ^-1^) and calculated by the differential equation:

$\frac{dC_{i}^{*}(t)}{\mathrm{dt}}=k_{D}\times({C_{\mathrm{ext}}\left( t \right)-C}_{i}^{*}(t))$ Eq. S1

The driving variable or model input is the external concentration *C*_ext_(t). The scaled internal concentration C_i_*(t) was linked to the stochastic death (SD) or individual tolerance (IT) model to describe the survival over time.

***Link to survival model – stochastic death***

In the SD model, a hazard rate is calculated following the differential equation

$\frac{\mathrm{dH}}{\mathrm{dt}}=k_{k}\times max(0,C_{i}^{*}\left( t \right)-z)$ Eq. S2

which describes hazard increasing in proportion to the exceedance of the threshold z (concentration). The killing rate *k*_k_ (concentration^-1^ time^-1^) is the proportionality constant.

The survival probability S_SD_(T) is the probability of individual to survive until time t and is calculated as:

$S_{SD}(t)=e^{-H(t)}\times e^{-h_{B}\times t}$ Eq. S3

where *h*_B_ (time^-1^) is the background hazard rate (estimated from control mortality).

***Link to survival model –individual tolerance***

In the IT model it is assumed that the threshold distribution is log-logistic. The survival probability for an individual is consequently calculated following a cumulative log-logistic distribution of the threshold z, given by [4]:

$F\left( t \right)=\frac{1}{1+\left( \frac{\max_{0\leq\tau\leq t} C_{i}^{*}\left( \tau\right)}{\alpha} \right)^{-\beta}}$ Eq. S4

where α is the median of the distribution of z (concentration), and β (-) is the shape parameter of the distribution. In the IT model, the survival is related to the maximum dose metric until time t rather than to the actual value of the dose metric at time t, because organisms that died previously remain dead. The probability to survive until time t is then:

$S_{IT}(t)=(1-F\left( t \right))\times e^{-h_{B}\times t}$ Eq. S5

## Calibration of model parameters

To find an optimal vector of parameter values Θ^opt^ for each of the SD and the IT models, parameter values were optimised with respect to the experimentally observed survival data. Survival probabilities for both the SD and the IT model are calculated in explicit dependence of the parameter vector $\theta$ and the external concentration over time, so formally

$S_{IT}\left( t \right)=S_{IT}\left( \theta,C_{ext}, t \right)=S_{IT}\left( (\alpha,\beta,k_{D},h_{B}),C_{ext}(t), t \right)$ Eq. S6

and

$S_{SD}\left( t \right)=S_{SD}\left( \theta,C_{ext}, t \right)=S_{SD}\left( (kk,z,k_{D},h_{B}),C_{ext}(t), t \right)$. Eq. S7

Survival data follow a multinomial distribution, hence the following likelihood function applies:

$\ln l\left( y | \Theta\right)= \sum_{i=1}^{n+1} (y_{i-1}-y_{i})\times ln(S_{i-1}\left( \Theta\right)-S_{i}\left( \Theta\right))$ Eq. S8

Equation S8 measures the goodness-of-fit of a survival model, given that *y_i_* are experimental observations at sampling times *i*, and *S_i_* are simulated survival probabilities at time *i*, given the parameter vector *Θ*. The log-likelihood function shows maximum values L^opt^ for an optimal fit between observed and simulated survival. In practice, usually the negative of the log-likelihood function, also called model error, is used because parameter estimation algorithms often minimise the objective function. The smaller the objective function, the better is the fit.

For this, the built-in optimisation routine *Simulated Annealing* of the method *NMinimize* of Mathematica (Wolfram Research, version 10.0) was used to obtain parameter sets for the best fit between data and model simulations by minimizing the negative of the log-likelihood function, i.e. maximize the log-likelihood function itself. Global stochastic optimiser algorithms like the used SimulatedAnnealing algorithm are supposed to deliver stable global optimal parameter sets. Convergence of the optimisation was checked by visual inspection of the fitted survival over time (figure ESM3.S1). Settings of the NMinimize method were: Method=SimulatedAnnealing, PerturbationScale=3, SearchPoints=50, PrecisionGoal = 8, MaxIterations = 20. For the numerical integration of the differential equations, the built-in ODE Solver NSolve was used with the settings Method=StiffnessSwitching, MaxStepSize =0.04, AccuracyGoal = 12.

Random numbers between 0 and 5 (between 0 and 0.5 for the background mortality) were used as starting values for parameter estimation. The optimization was repeated three times, where for the second and third optimization run random numbers between 0 and the ceiling of the optimal value of the optimization run before were used as starting values. There were no constraints on the parameter intervals, only they had to be positive (> 0). No further assumptions were made to identify the optimal parameter values.

The optimisation routine yielded an optimal parameter vector *Θ^opt^* for which the log-likelihood function shows the highest values $\ln l\left( y | \Theta^{opt} \right)$ given the data set *y.* Optimisation was repeated two times after the first optimisation run, each time using the optimum values from the optimisation step before as starting values, finally resulting in an optimal parameter set *Θ^opt^* for each species and model version. All parameter vectors *Θ* being tested during the optimisation routine have been stored together with the resulting likelihood values $\ln l\left( y | \theta\right)$ for further analyses.

## Numerical estimation of parameter confidence intervals

The likelihood ratio method was used to estimate confidence intervals for the optimal parameter *Θ^opt^* (Meeker & Escobar, 1995; Vugrin et al., 2007). Confidence intervals for the single parameters for a given confidence level *α* were calculated as those parameter values, for which the likelihood ratio fulfils the condition

$-2\left[ \ln l\left( y | \Theta\right)-\ln l\left( y | \Theta^{opt} \right) \right] \leq\chi_{df,1-\alpha}^{2}$ Eq. S9

with $\chi_{df,1-\alpha}^{2}$ being the value of the Chi-square-distribution for the confidence level α, and the degrees of freedom of the likelihood ratio (for single parameter confidence intervals df=1). To find these parameter values, one value in the parameter vector, say parameter Θ_i_, was successively decreased, starting at the maximum-likelihood estimate, i.e. the best parameter value. All parameter values with exception of parameter i were again optimised (using the NMinimize method and the SimulatedAnnealing algorithm in Mathematica) to give a best fit to the experimental data. The value $\ln l\left( y | \Theta\right)$ of the log-likelihood function corresponding to the parameter vector $\Theta$= ($\Theta_{1}^{\mathrm{opt}},\Theta_{2}^{\mathrm{opt}},\ldots,\Theta_{i}, \ldots,\Theta_{n}^{\mathrm{opt}})$ was calculated. This procedure was repeated until the value of $\ln l\left( y | \Theta\right)$satisfied equation S9. Likewise, this procedure was repeated for successively increasing parameter values, again starting with the optimal maximum-likelihood estimate. The first parameter values that did not fulfil condition (eq. S9) were taken as lower and upper confidence interval limits to the confidence level α. This procedure was performed for all parameters and α=0.95.

## Model predictions and calculation of model performance statistics

Next to biological variability , the main sources of uncertainty in model predictions are i) the stochasticity of the survival process in small cohorts of individuals, and ii) the uncertainty of the estimated model parameters. These uncertainties can be quantified and hence taken into account. We followed the approach as outlined in Ashauer et al. (2016). For convenience, the methodology is repeated here.

### Stochasticity of survival and probabilistic modelling of survival over time

Survival is a binary process, i.e. an individual can be either alive or death. The number of individuals in a group of individuals influences the uncertainty of model predictions about the survival of the group of individuals, especially for small numbers (n<100) such as in the current experiments. Therefore, survival from the pulsed exposure profiles as used in experiments MS_V and MC_V was modelled as a binomial process, with the number of surviving individuals being proportional to the conditional binomial distribution

$Y_{t+\Delta t}\mathcal{\sim B(}p_{t+1},Y_{t})$ Eq. S10

where$Y_{t+\Delta t}$is the number of survivors in a population at time $t+\Delta t$, *Y_t_* is the number of individuals being alive at time $t$, and $p_{t+\Delta t}$ is the conditional probability to survive from time $t$ until time $t+\Delta t$, given as

$p_{t+\Delta t}=\frac{S(\theta,C_{ext},t+\Delta t)}{S(\theta,C_{ext},t)}$ Eq. S11

where$S(\theta,C,t)$ is the survival probability calculated by the SD or the IT model for the parameter vector $\theta$, the external concentration time course $C_{ext}$, and the time point $t$. Survival probability is the probability to survive until time t, which is different to the conditional probability of eq. (S11). Note that time steps are not fixed to 1 here, as the survival probabilities can be evaluated for arbitrary small or large time steps, hence conditional probabilities can be modelled as a random process using a flexible time step $\Delta t$.

For a parameter vector $\theta$, an external concentration regime *C_ext_* and an initial population size *Y_0_*, the number of surviving individuals in the population is modelled in an iterative way, starting with *Y_0_* individuals at t=0, by drawing for every new time step $t+\Delta t$ from a binomial distribution parameterised by the number of living organisms at time $t$, and the calculated survival rates for times $t$ and $t+\Delta t$ as given in equations S10 and S11. By performing *n_Smax_* repetitions of this procedure, we obtain *n_Smax_* realisations of the survival probabilities within a population of initial size *Y_0_*, given the parameter vector $\theta$ and the external exposure *C_ext_*, from which statistical descriptors such as median and percentiles are calculated.

### Including parametric uncertainty in forecasts

Parametric uncertainty in model simulations is in addition to the stochasticity of survival itself. To consider parameter uncertainties, the following approach is used.

To approximate the joint confidence regions for the parameters, a number of *n_Umax_* parameter sets from the confidence region $\Theta_{conf}$ are drawn (critical value in a likelihood ratio test = 7.81473 from chi-square distribution, df=3, p=0.05), formally expressed as

$\Theta_{conf}=\left\{ \theta\right|-\ln l\left( y | \theta\right) \leq-ln l\left( y | \theta^{opt} \right)\cdot\frac{\chi_{df,1-\alpha}^{2}}{2}\}$ Eq. S12

The degrees of freedom for the likelihood ratio that is being used for the construction of the joint confidence region are defined by the difference in free model parameters, that is in this case df=3, because for optimisation all three model parameters of the GUTS-SIC-SD or GUTS-SIC-IT models were free, whereas for the construction of the three-dimensional joint confidence region all model parameters were fixed. The background mortality rate constant was not taken into account here, because it was not considered for the uncertainty calculations. For every drawn parameter vector, survival over time is simulated performing n_Smax_ repetitions to account for the stochasticity of survival per exposure profile, and the results are. The total numbers of simulations hence amounts to *n_max_* = *n_Umax *_ n_Smax_*. These *n_max_* realisations of the simulated numbers of surviving individuals within the population of initial size *Y_0_* is described statistically by the median and upper and lower percentiles of the distribution of the numbers of survivors, quantifying uncertainty in the model predictions. For this study, we used *n_Smax_*=10, *n_Umax_*=1,000, hence performed *n_max_*=10,000 simulations of survival over time per exposure scenario .

### Dose response curves for pulsed exposures

Simulated dose response relationships for the pulsed exposure profiles as tested experimentally in experiments MS_V and MC_V were constructed by prediction of survival over time for a series of manipulated pulsed exposure profiles. The concentrations as measured in the original pulsed exposure experiments were multiplied with a set of 61 multiplication factors, ranging from 10^-2.5^ to 10^3.5^. Predictions were done both deterministic (using the optimal parameter vector) and probabilistic (with 10000 Monte Carlo runs). Simulated survival at the end of the pulsed experiments as appearing from the optimal parameter sets, and median, and the 10^th^ and 90^th^ percentiles as obtained from the probabilistic simulations together with the exposure concentrations generated a series of data points that were interpolated to create the dose response curves corresponding to the last day of the respective experiments.

### Calculation of model performance statistics

A set of indicators for the accuracy of the model predictions were calculated for the survival over time. The model prediction error (MPE) (Nyman et al., 2012) was calculated defined as

$\frac{1}{n}\sum\frac{\left| S_{obs}-S_{model} \right|}{S_{model}}$, Eq. S13

where n is the number of data points per experiments, and S are the fractions of observed and modelled survivors. Additionally, the correlation between observed and predicted survival probabilities per time step is calculated per data set, where the survival probability between time t and the next observed time point t+1 is defined simply as the ratio *S(t+1)/S(t)*.

A second criterion is based on the expectation that predicted and observed survival numbers match the 1:1 line in a scatter plot. The classical root-mean-square error (RMSE) aggregates the magnitude of the prediction errors for various time-points into a single measure of predictive power. In order to provide a criterion expressed as a percentage, the RMSE normalised by the mean of the observations is used:

$NRMSE=\frac{RMSE}{\bar{Y}}=\frac{1}{\bar{Y}}\sqrt{\frac{1}{n}\sum_{i=1}^{n} \left( y_{obs,i}-y_{pred,i} \right)^{2}}$ Eq. S14

where $\bar{Y}=\frac{1}{n}\sum_{i=1}^{n} y_{obs,i}$ is the mean of the *n* observed numbers of survivors $y_{obs,i}$ for i=1,…,n. Numbers $y_{pred,i}$ correspond to the median of the predicted numbers of survivors at each time-point.

A third criterion for the model accuracy at the end of the experiments was developed based on the survival probabilities. The probability to survive an exposure profile from the beginning to the end of an experiment is given as ratio between the number of surviving (Y_obs,tend_) and the number of initial individuals (Y_init_) in a test, hence the difference between the observed and modelled survival probabilities, or in other words the survival probability prediction error (SPPE) is given as

$SPPE=\frac{Y_{obs,tend}}{Y_{init}}-\frac{Y_{modelled,tend}}{Y_{init}}*100=\frac{Y_{obs,tend}-Y_{modelled,tend}}{Y_{init}}*100$ Eq. S.15

The SPPE is suggested as indicator of model accuracy considering survival probabilities only at the end of a tested exposure profile. The SPPE indicator is negative (between 0 and -100%) for an underestimation of effects, and positive (between 0 and 100%) for an overestimation of effects. An SPPE value of 0% means an exact prediction of the observed survival probability at the end of the experiment.
