# Supplementary material for: Calibration and validation of toxicokinetic-toxicodynamic models for three neonicotinoids and some aquatic macroinvertebrates
Source: Ecotoxicology. 2018 May 1;27(7):992–1007. doi: 10.1007/s10646-018-1940-6 (PMC6132984; doi:10.1007/s10646-018-1940-6)
Supplement: Supplementary file 3 — additional figures [file 10646_2018_1940_MOESM3_ESM.docx]

| **I MS_C1** | 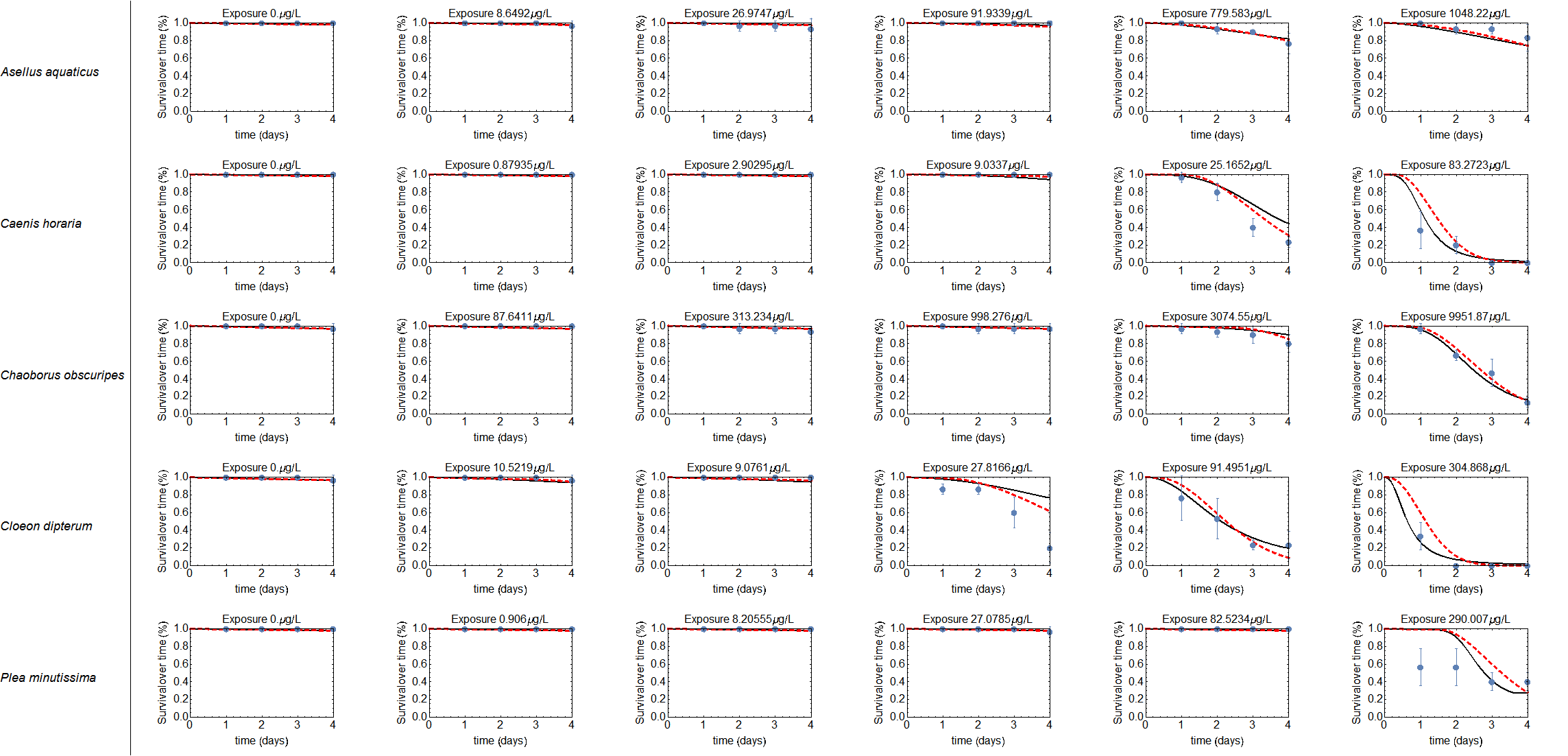 |
| --- | --- |
| **II MC_C1** | 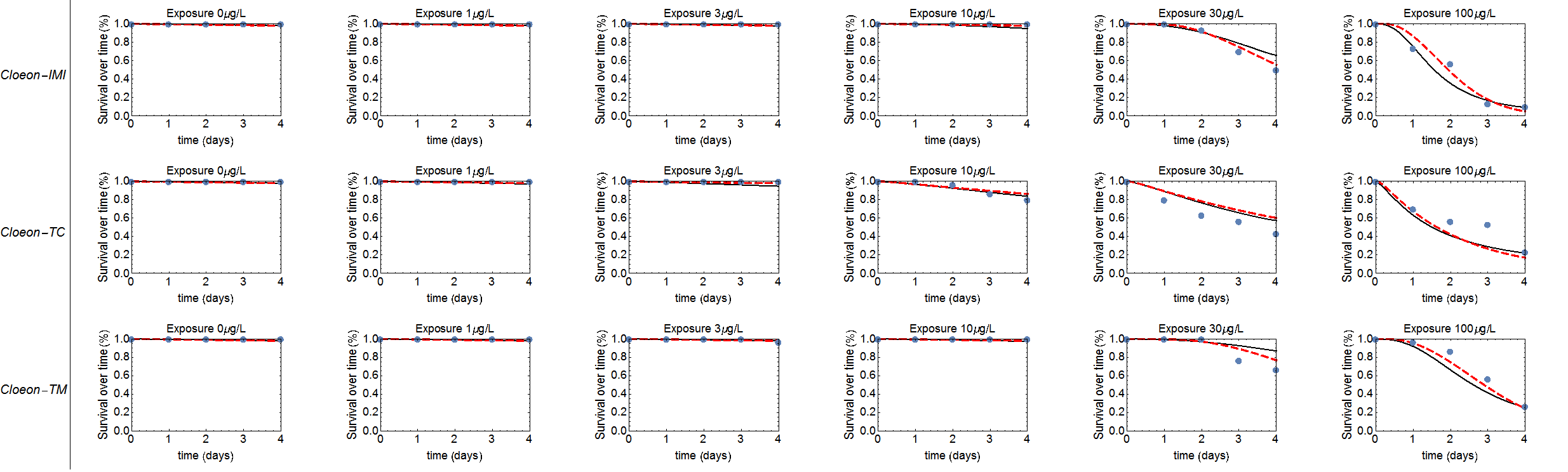 |
| **III**  **MC_C2** | 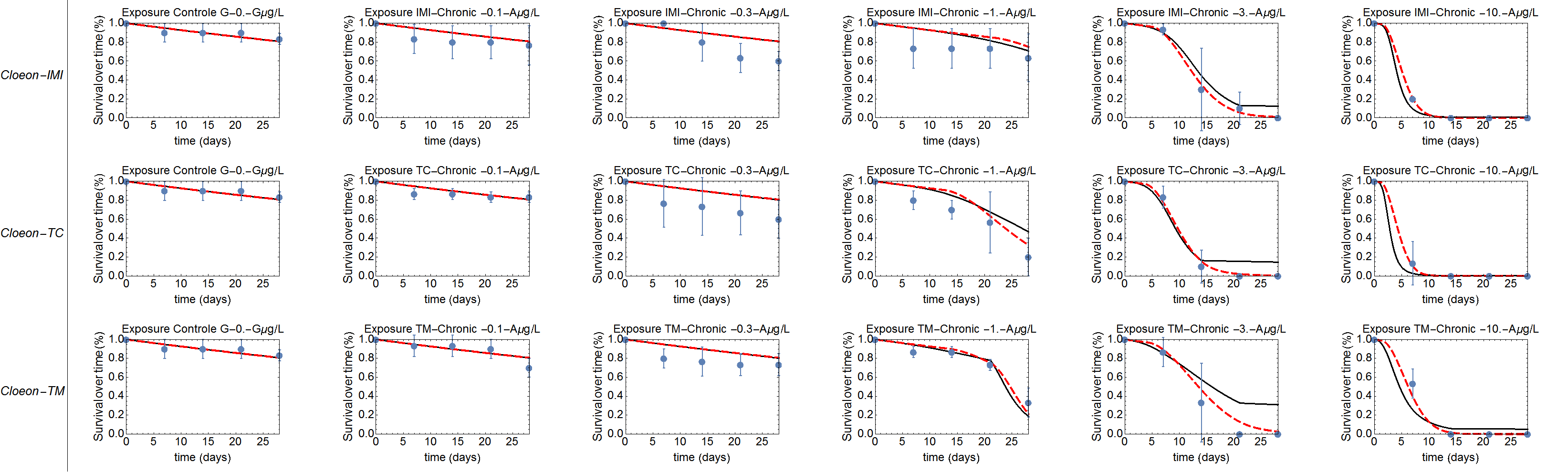 |

**Figure S1**: Plots of observed survival data (symbols) and fitted models (red dashed lines: SD model; black solid lines: IT model) over time for all calibration data sets. Exposure levels are given in the titles of the single plots.


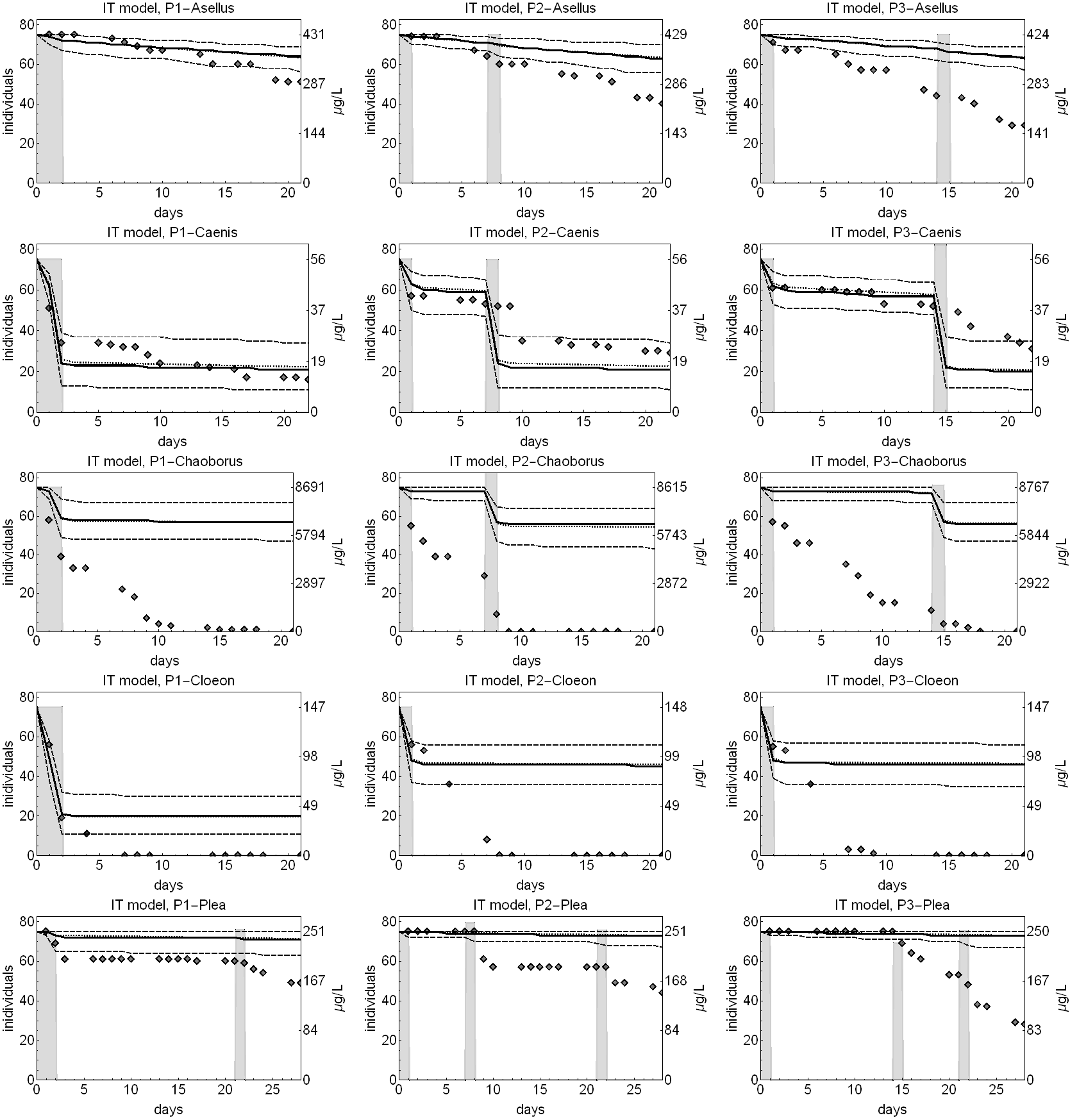


**Figure S2:** Observed and modelled survival of individuals of 5 macroinvertebrate species under 3 pulsed exposure profiles for imidacloprid, predicted based on acute testing (exp. MS_C1) using the IT model. Diamonds indicate observed numbers of living organisms (exp. MS_V). The dashed lines show the 5 and 95 percentile of 10.000 probabilistic simulations of the time course of survival (ref. section SI-1.2.3.), the solid line is the respective median. Black dotted lines show the predicted deterministic rate of survivors. Grey bars show the exposure levels over time, concentrations as indicated in the axes labels on the right hand side of the single panels.


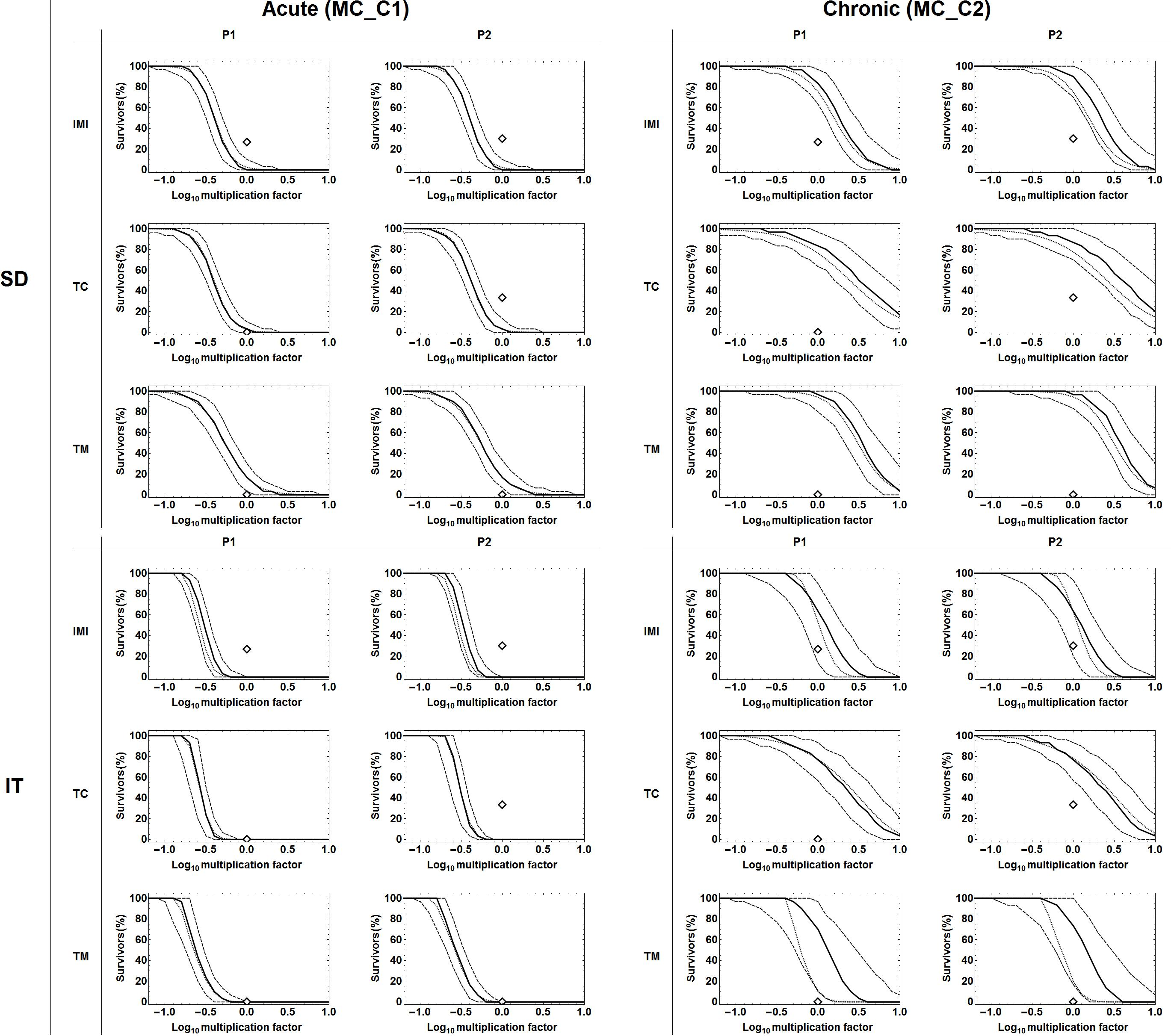


**Figure S3**: Predictions of experiment MC_V (survival of *C. dipterum* under exposure to imidacloprid (IMI), thiacloprid (TC), thiametoxam (TM)) with the SD (top rows) and the IT model (bottom rows), calibration based on data from acute experiments (MC_C1, left columns or chronic experiments (MC_C2, right columns). Diamonds indicate observed numbers of living organisms (exp. MC_V). The dashed lines shown the 5 and 95 percentile of 10.000 probabilistic simulations of the survival at the end of the experimental testing (ref. section SI-1.2.3.), the solid line is the respective median. Black dotted lines show the deterministic rate of survivors.
