# Supplementary material for: Calibration and validation of toxicokinetic-toxicodynamic models for three neonicotinoids and some aquatic macroinvertebrates
Source: Ecotoxicology. 2018 May 1;27(7):992–1007. doi: 10.1007/s10646-018-1940-6 (PMC6132984; doi:10.1007/s10646-018-1940-6)
Supplement: Supplementary file 4 — additional tables [file 10646_2018_1940_MOESM4_ESM.docx]

# Tables

**Table S1**: Treatments in experiments MC_C2 and MC_V

| Compound | *imidacloprid* | *thiacloprid* | *thiamethoxam* |  |  |
| --- | --- | --- | --- | --- | --- |
|  | (Sigma-Aldrich, >99%) | (Ehrenstorfer, 99.2%) | (Ehrenstorfer, 98.5%) |  | dosing |
| Experiment | **Treatment level (µg/l)** | | | **Replication** | t (days) |
| MC_C2 | Controls | Controls | Controls | n= 9 | 0 |
|  | 0.1 | 0.1 | 0.1 | n= 3 | 0 |
|  | 0.3 | 0.3 | 0.3 | n= 3 | 0 |
|  | 1 | 1 | 1 | n= 3 | 0 |
|  | 3 | 3 | 3 | n= 3 | 0 |
|  | 10 | 10 | 10 | n= 3 | 0 |
| MC_V, P1 | 70 | 35 | 85 | n= 3 | 0, 7 |
| MC_V, P2 | 70 | 35 | 85 | n= 3 | 0, 14 |

**Table S2**: Parameter values and confidence levels

| Data set | Com-pound | SD model | LL^a^ | lcl^b^ | k_D_ | ucl^b^ | lcl^b^ | k_k_ | ucl^b^ | lcl^b^ | z | ucl^b^ |
| --- | --- | --- | --- | --- | --- | --- | --- | --- | --- | --- | --- | --- |
| *MS_C1* | *IMI* | *A. aquaticus* | -64.2819 | - | 2.5E-4 | 3.9E-2 | - | 1.3E-1 | 2.3E+1 | - | 0.0E+0 | - |
| *MS_C1* | *IMI* | *C. horaria* | -55.3914 | 1.5E-3 | 9.5E-2 | 4.6E-1 | 3.2E-2 | 1.4E-1 | 6.3E-1 | 1.5E-1 | 2.5E+0 | 7.5E+0 |
| *MS_C1* | *IMI* | *C. obscuripes* | -83.2063 | 2.6E-4 | 1.9E-3 | 1.2E-2 | 3.0E-3 | 1.9E-2 | 9.0E-2 | 1.9E+0 | 1.5E+1 | 6.5E+1 |
| *MS_C1* | *IMI* | *C. dipterum* | -93.3394 | 2.6E-4 | 2.6E-2 | 1.3E-1 | 2.3E-2 | 1.5E-1 | 8.2E-1 | 1.6E-1 | 7.9E-1 | 3.4E+0 |
| *MS_C1* | *IMI* | *P. minutissima* | -23.8894 | 2.1E-2 | 2.9E-1 | 1.8E+1 | 2.4E-4 | 1.7E-3 | 3.7E-2 | 5.6E+0 | 7.1E+1 | 2.5E+2 |
| *MC_C1* | *IMI* | *CD –acute* | -28.8175 | 3.3E-3 | 1.8E-1 | 3.4E+0 | 2.7E-4 | 3.1E-2 | 1.7E-1 | 9.4E-2 | 4.5E+0 | 1.4E+1 |
| *MC_C1* | *TC* | *CD –acute* | -40.7011 | 8.5E-2 | 7.1E+0 | 6.4E+2 | 2.4E-3 | 4.7E-3 | 1.5E-2 | 3.3E-3 | 2.9E+0 | 9.5E+0 |
| *MC_C1* | *TM* | *CD –acute* | -26.9902 | 7.8E-4 | 1.6E-3 | 4.5E-2 | 1.3E-2 | 1.4E+0 | 7.2E+0 | 5.6E-4 | 5.7E-2 | 1.7E-1 |
| *MC_C2* | *IMI* | *CD –chronic* | -170.03 | 3.2E-3 | 4.2E-2 | 1.1E-1 | 7.2E-2 | 2.6E-1 | 7.7E-1 | 1.2E-1 | 5.4E-1 | 1.3E+0 |
| *MC_C2* | *TC* | *CD –chronic* | -167.871 | 5.0E-3 | 2.8E-2 | 5.6E-2 | 2.7E-1 | 5.5E-1 | 6.7E+0 | - | 2.6E-1 | 4.3E-1 |
| *MC_C2* | *TM* | *CD –chronic* | -171.581 | 1.1E-3 | 4.1E-3 | 4.1E-2 | 1.3E-1 | 1.3E+0 | 5.9E+0 | - | 5.0E-2 | 2.1E-1 |
|  |  | **IT model** | **LL^a^** | **lcl**^b^ | **k_D_** | **ucl**^b^ | **lcl**^b^ | **α** | **ucl**^b^ | **lcl**^b^ | **β** | **ucl**^b^ |
| *MS_C1* | *IMI* | *A. aquaticus* | -67.114 | 4.6E-5 | 5.0E-4 | 1.7E-3 | 5.7E-1 | 4.2E+0 | 3.9E+1 | 8.2E-1 | 1.6E+0 | 3.1E+0 |
| *MS_C1* | *IMI* | *C. horaria* | -64.734 | 2.8E-4 | 3.0E-3 | 8.2E-2 | 1.4E-1 | 2.8E-1 | 6.6E-1 | 2.3E+0 | 3.3E+0 | 4.9E+0 |
| *MS_C1* | *IMI* | *C. obscuripes* | -85.15 | 1.0E-3 | 2.0E-3 | 9.4E-3 | 2.4E+1 | 5.1E+1 | 1.1E+2 | 2.2E+0 | 3.6E+0 | 5.4E+0 |
| *MS_C1* | *IMI* | *C. dipterum* | -114.883 | - | 1.4E-2 | 4.7E-2 | 5.8E-1 | 2.7E+0 | 5.7E+0 | 1.8E+0 | 2.3E+0 | 3.1E+0 |
| *MS_C1* | *IMI* | *P. minutissima* | -31.573 | 1.3E-1 | 5.5E-1 | 1.5E+0 | 1.1E+2 | 2.9E+2 | 4.3E+2 | 2.3E+0 | 6.5E+0 | 3.0E+1 |
| *MC_C1* | *IMI* | *CD –acute* | -29.8106 | 6.9E-7 | 1.7E-3 | 1.5E-1 | 6.8E-2 | 2.7E-1 | 2.1E+0 | 1.2E+0 | 2.5E+0 | 4.4E+0 |
| *MC_C1* | *TC* | *CD –acute* | -41.1032 | 1.7E-4 | 4.1E-3 | 3.0E-1 | 3.3E-2 | 6.2E-1 | 3.5E+1 | 6.5E-1 | 1.3E+0 | 2.0E+0 |
| *MC_C1* | *TM* | *CD –acute* | -29.4407 | 4.6E-4 | 4.5E-3 | 9.8E-2 | 8.7E-3 | 1.2E+0 | 7.0E+0 | 1.3E+0 | 2.6E+0 | 4.3E+0 |
| *MC_C2* | *IMI* | *CD –chronic* | -174.501 | 1.9E-4 | 3.8E-4 | 1.2E-3 | 7.7E-3 | 1.5E-2 | 8.8E-2 | 2.1E+0 | 4.1E+0 | 6.2E+0 |
| *MC_C2* | *TC* | *CD –chronic* | -182.411 | 8.5E-4 | 1.7E-3 | 8.5E-3 | 1.7E-2 | 4.2E-2 | 1.9E-1 | 2.0E+0 | 4.0E+0 | 6.0E+0 |
| *MC_C2* | *TM* | *CD –chronic* | -186.249 | 1.3E-4 | 2.5E-4 | 7.8E-4 | 4.8E-3 | 1.2E-2 | 1.3E-1 | 1.3E+0 | 2.6E+0 | 3.9E+0 |

^a^ LL: log-likelihood value  ^b^ lcl: lower confidence limit, ucl: upper confidence limit, 90% confidence level. -: could not be calculated since the algorithm did not converge.
